# Genome sequence of the model sulfate reducer *Desulfovibrio gigas*: a comparative analysis within the *Desulfovibrio* genus*

**DOI:** 10.1002/mbo3.184

**Published:** 2014-07-23

**Authors:** Fabio O Morais-Silva, Antonio Mauro Rezende, Catarina Pimentel, Catia I Santos, Carla Clemente, Ana Varela–Raposo, Daniela M Resende, Sofia M da Silva, Luciana Márcia de Oliveira, Marcia Matos, Daniela A Costa, Orfeu Flores, Jerónimo C Ruiz, Claudina Rodrigues-Pousada

**Affiliations:** 1 Instituto de Tecnologia Quómica e Biológica – Antonio Xavier, Universidade Nova de Lisboa (ITQB-UNL)Av. da República – Estação Agronómica Nacional, 2780-157, Oeiras, Portugal; 2 Grupo Informática de Biossistemas, Centro de Pesquisa René Rachou – FIOCRUZBelo Horizonte, Minas Gerais, Brazil; 3 STAB VIDA - Madan Parque Rua dos Inventores s/sala 2.182825-182, Caparica, Portugal; 4 Departamento de Microbiologia, Programa de Pós-Graduação em Bioinformática, Universidade Federal de Minas GeraisBrazil

**Keywords:** Analysis, comparative genomics, *Desulfovibrio gigas*, genome

## Abstract

*Desulfovibrio gigas* is a model organism of sulfate-reducing bacteria of which energy metabolism and stress response have been extensively studied. The complete genomic context of this organism was however, not yet available. The sequencing of the *D. gigas* genome provides insights into the integrated network of energy conserving complexes and structures present in this bacterium. Comparison with genomes of other *Desulfovibrio* spp. reveals the presence of two different CRISPR/Cas systems in *D. gigas*. Phylogenetic analysis using conserved protein sequences (encoded by *rpo*B and *gyr*B) indicates two main groups of *Desulfovibrio* spp, being *D. gigas* more closely related to *D. vulgaris* and *D. desulfuricans* strains. Gene duplications were found such as those encoding fumarate reductase, formate dehydrogenase, and superoxide dismutase. Complexes not yet described within *Desulfovibrio* genus were identified: Mnh complex, a v-type ATP-synthase as well as genes encoding the MinCDE system that could be responsible for the larger size of *D. gigas* when compared to other members of the genus. A low number of hydrogenases and the absence of the *codh/acs* and *pfl* genes, both present in *D. vulgaris* strains, indicate that intermediate cycling mechanisms may contribute substantially less to the energy gain in *D. gigas* compared to other *Desulfovibrio* spp. This might be compensated by the presence of other unique genomic arrangements of complexes such as the Rnf and the Hdr/Flox, or by the presence of NAD(P)H related complexes, like the Nuo, NfnAB or Mnh.

## Introduction

Sulfate-reducing bacteria (SRB) are probably one of the most ancient forms of life on Earth. This group of anaerobic microorganisms, widespread in anoxic habitats, uses sulfate as main terminal electron acceptor to degrade organic compounds, with the consequent production of sulfide (Muyzer and Stams 2008). This process is extremely important in the sulfur and carbon cycles, since ∼50% of the organic carbon mineralization in marine sediments is due to sulfate reduction (Jorgensen 1982). SRB are metabolically very versatile microorganisms, being able to use organic and inorganic substrates, as well as other short-chain fatty acids or ethanol for sulfate reduction. In recent years, new species were found to be able to grow on more diverse and less degradable substrates such as hydrocarbons or aromatic compounds (Rabus et al. 2006). Furthermore, due to the fact that many SRB use H_2_ as an important substrate for sulfate reduction, they are able to participate in interspecies hydrogen transfer processes in synthropic communities with archaea (Walker et al. 2009; Plugge et al. 2010; Li et al. 2011). As a result of their metabolic flexibility, SRB can be found in almost all ecological environments on the planet. Moreover, these bacteria possess a wide biotechnological potential, especially in bioremediation of sulfate and heavy metals from natural environments and in removal of industrial waste liquids and sewage (Janssen et al. 2001; Lenz et al. 2008). On the other hand, due to the production of high amounts of hydrogen sulfide, SRB have large negative economic impact mainly as causative agents of microbial corrosion processes in anaerobic environments like those occurring in offshore oil production or waterlogged clay soils, resulting in economic losses (Hamilton 1985). Furthermore, they can create problems through a change in oil composition and souring of petroleum reservoirs (Huang and Larter 2005; Vance and Thrasher 2005).

Recent advances in genomics, biochemistry, and genetics of the SRB have greatly helped to identify the essential enzymes and complexes that participate in sulfate respiration. The reduction of sulfate to sulfide during the respiratory process occurs in the cytoplasm. As such, electron transport chains and carriers must provide a link for the flow of the reducing equivalents ([H^+^] and electrons) between dehydrogenases and the terminal reductases (Rabus et al. 2006). Despite many efforts to understand the sites and mechanisms of energy conservation in sulfate respiration, the electron-transfer pathways that generate ATP from oxidative phosphorylation and create a proton gradient are not yet fully understood (Pereira et al. 2011). Most of the studies are focused on understanding the principles of sulfate reduction using *Desulfovibrio* genus. Among the various members of this genus, *Desulfovibrio gigas,* a curved rod bacterium, whose name was inspired by its unusual size (up to 11 *μ*m) was for the first time isolated in 1963 by Jean LeGall from a water pond (LeGall 1963). After its isolation, this bacterium was used by many different groups to elucidate the structure of enzymes participating in energy transfer reactions such as hydrogenases, formate dehydrogenases, ferredoxins, cytochromes, and the xantine oxidase-related aldehyde oxido-reductase (molybdenum-containing aldehyde oxido-reductase; MOP) (Ambler et al. 1969; Romao et al. 1995; Volbeda et al. 1995; Matias et al. 1996; Frazao et al. 2000; Raaijmakers et al. 2002; Hsieh et al. 2005). Mechanistic and functional processes related to the energy metabolism and stress response have been also well studied in *D. gigas* (Silva et al. 2001; Broco et al. 2005; Rodrigues et al. 2006a; Morais-Silva et al. 2013). However, despite the accumulated information about this bacterium, a clear whole-genome context of the genes and metabolic complexes is not yet available for *D. gigas*. Previous analyses and comparison between the different species of SRB revealed that the composition of energy metabolism proteins, as well as stress-related proteins can vary quite significantly (Rabus et al. 2006; Pereira et al. 2008, 2011). *D. gigas* may, therefore, react to environmental cues and adapt to different environments by using different metabolic and structural components. Genome sequencing analysis is an important tool in order to fully understand which components may be involved in these adaptation and survival mechanisms. In this article, we examine the whole-genome sequence of this organism and perform a comparative genomic analysis with other *Desulfovibrionaceae*.

## Materials and Methods

### DNA sequencing, assembly, and annotation

DNA was isolated with the Wizard Genomic DNA Purification Kit (Promega, Mannheim, Germany). Sequencing was performed using a combination of several approaches: Sanger sequencing, using small fragment (2–6 kb) libraries; High throughput Roche Diagnostics 454 GS20 sequencing (Roche Diagnostics, Mannheim, Germany) (Keygene) and Illumina′s Solexa sequencing technology. Final gap closure was obtained either by primer walking or resequencing in the Personal Genome Machine (PGM) platform set up in STAB VIDA. The global coverage was 159.68-fold sequences. Ab initio assembly was performed using Velvet version 0.7.55 software (Zerbino and Birney 2008), and the consensus genomic sequence was obtained with Phrap (http://www.phrap.org/phredphrapconsed.html).

Structural annotation was performed using FgenesB (http://www.softberry.com), RNAmmer (Lagesen et al. 2007), tRNA-scan-SE (Lowe and Eddy 1997) and Tandem Repeat Finder (tandem.bu.edu/trf/trf.html). Functional annotation was performed by similarity, using public databases and InterProScan analysis (Zdobnov and Apweiler 2001). Protein-coding sequences were manually curated using Artemis (Rutherford et al. 2000). Comparative analyses for *Desufovibrio* spp. were performed using the BLAST–NCBI (Altschul et al. 1990) and InterProScan databases. The genomic and plasmidic sequences of *D. gigas* ATCC19364 were submitted to GenBank under the Acession No. CP006585 and CP006586, respectively.

### Phylogenetic analysis

Evolutionary relationship between *Desulfovibrio* species was constructed using RpoB and GyrB concatenated sequences downloaded from GenBank (http://ftp://ftp.ncbi.nlm.nih.gov/). Sequence alignment was done using MAFFT software (Katoh et al. 2002) and LG evolutionary model (Le and Gascuel 2008) was selected for analysis using the ProtTest version 2. (Abascal et al. 2005). PhyML version 3.0 algorithm and the Maximum Likelihood method (Guindon et al. 2010) were used to create the phylogenetic tree. The evolutionary history of Cas1 proteins was inferred by using the Maximum Likelihood method based on the JTT matrix-based model (Jones et al. 1992).

In order to assess the number of genes shared between *D. gigas* and other *Desulfovibrio* species, a Venn diagram was built using the EDGAR database (https://edgar.computational.bio.uni-giessen.de/).

### Codon usage analysis

The *D. gigas* codon usage was determined by using an EMBOSS tool called *cusp* (Rice et al. 2000). This program calculates a codon usage table for one or more nucleotide coding sequences (Table S2). The codon usage table and *D. gigas* coding sequences were next used in another EMBOSS program called *cai*, which calculates the Codon Adaptation Index (CAI) (Sharp and Li 1987).

### Protein interaction network

A list of *D. gigas* proteins related to chemotaxis (Table S6) and oxygen response (Table S7) were used as query in the search against the EDGAR database. The number of *D. gigas* orthologs found in 13 *Desulfovibrio* strains is depicted in radar graphs.

## Results and Discussion

### General genome features

**Figure 1:**
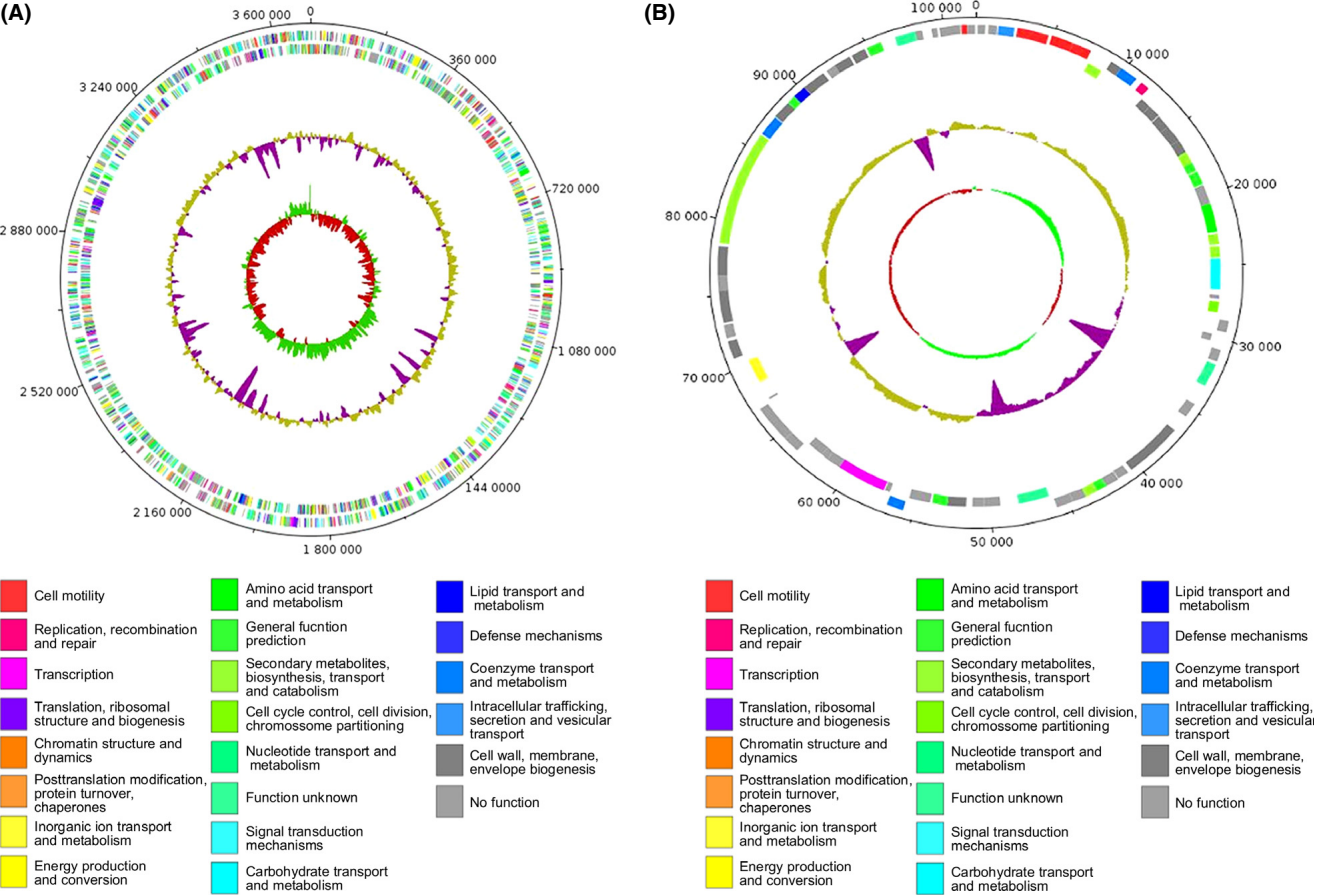
Structural representation of the circular chromosome (A) and plasmid (B) of *Desulfovibrio gigas*. Circular representations, from inside to the outside represent: (i) GC skew, richness of guanine over cytosine in the positive strand represented in green and cytosine over guanine represented in red; (ii) GC content, below average in purple, above average in gold; (iii) positive strand coding regions (below) and negative strand coding regions (above) colored according to COG functional terms of the best hit obtained from *Blastp* program; (iv) nucleotide position indicated in circular scale.

The genome of *D. gigas* (CP006585) consists of one circular chromosome of 3,693,899 base-pairs (bp) having 3370 genes of which 3273 are protein-coding (see Table1 and Fig1A), classified according to its predicted COG function (Table S1). The genome has a G+C content of 63.4% that reflects a biased codon usage. Indeed, *D. gigas* prefers high G+C codons (66.87%), with a clear preference for cytosine (C) in the 3rd position (82.03% and Table S2). Indeed, among synonymous codons used by the 20 aminoacids, 13 of them are using more frequently one triplet ending by C. There are however, a few exceptions as leucine and valine which use respectively the CTG and GTG. The CAI calculated for all coding sequences has an average of 0.663, with a maximum of 0.863 (DGI_1104, a putative hydrolase) and the minimum of 0.198 (DGI_2086 and 3377, both hypothetical proteins). Genes encoding the hydrogenases (Table S24), as well as energy conserving transmembrane complexes (Table S25) present a higher CAI value, around 0.701, than the average, suggesting that these genes have a higher expression potential which was experimentally shown in the case of both the hydrogenases Ech and Hyn, (Morais-Silva et al. 2013).The genome is very compact as observed by its gene density of 1128 bp per gene and the average length of each gene is 993 bp. It contains 17 transposases (Table S3), whereas in other SRB genomes this number is in average 34 (Bennett 2004). This relative low number of transposable elements in *D. gigas* may indicate a low rate of reorganization of its genome. Other features include 47 pseudogenes and 48 tRNAs (Table1), as well as 9 selenocysteine containing proteins (Table S4). Surprisingly, one single operon of rRNA was found in *D. gigas* in contrast to what was detected in other *Desulfovibrio* spp. that contain between 3 and 6 operons. The recently sequenced genome of the new strain *Salinarchaeum* sp. HArcht-Bsk1T, also contains one single rRNA operon (Dominova et al. 2013) as well as the bacterium *Mycobacteria,* a fact that was associated to their slow growth (Arnvig et al. 2005). The high generation time of *D. gigas* of around 8 h may be as well related to this fact. Besides, having in the genome solely 17 genes encoding transposases and only a single rRNA operon, may also indicate a decreased genome rearrangement, as multiple rRNA operons serves as sites for homologous recombination (Helm et al. 2003).

**Table 1:** General genome features of *Desulfovibrio gigas*.

Features	Value	% of total
Genome		
Genome size (bp)	3,693,899	100
DNA coding region (bp)	3,249,714	87.98
G + C content (bp)	2,341,530	63.39
Extracromossomal elements	1	
Number of replicons	1	
Total number of genes	3370	100
Stable rRNAS		
rRNAs	3	0.09
tRNAs	48	1.42
Protein-coding genes	3273	97.09
Genes density (bp/gene)	1128	
Average length of a gene (bp)	993	
Pseudogenes	47	1.39
Genes with assigned COG	2273	67.45
Selenocysteine-containing proteins	9	
Genes without assigned function	999	29.64
Poorly chracterized genes	395	11.72
Other elements		
CRISPR repeats	6	
Cas operons	2	
Transposases	17	
Mobile elements	1	

The plasmid of this bacterium (CP006586) has a size of 101,949 bp, containing 75 ORFs, of which 72 are coding regions (Table2 and Fig1B). Approximately one-third of the encoded polypeptides are annotated as hypothetical. Regarding the remaining annotated ORFs, the most representative functional group is composed of 12 proteins encoding acetyl, methyl, and glycosyl transferases. Interestingly, we could also identify an operon of 12 ORFs (DGIp_00010-00120) encoding a type II secretory system (T2SSs) which is involved in the secretion of folded and/or oligomeric exoproteins (Douzi et al. 2012). We have also identified a 30 kb operon encoding a set of capsule polysaccharide biosynthesis (*kps*) and transporter (*tag*) proteins. These features may indicate a mechanism used by *D. gigas* to secret and transport folded exoproteins. Another remarkable feature of *D. gigas* plasmid is related to the presence of the *apsK* gene encoding a bi-functional protein, predicted to have a sulfate adenylyltransferase and adenylylsulfate kinase activities (Marchler-Bauer et al. 2013).

**Table 2:** General plasmid features of *Desulfovibrio gigas*.

Features	Value
Size (bp)	101,949
G + C contente (bp)	64,081
DNA coding region (bp)	79,425
Pseudogenes	3
Protein-coding genes	72
Gene density (bp/gene)	1415
Average length of a gene (bp)	1103

### Desulfovibrio gigas and its size

The size of *Desulfovibrio gigas* is larger than the one of other *Desulfovibrio* spp. Its length is of 5–10 *μ*m and the width of 1.2–1.5 *μ*m, whereas the other species have a cell size of 3–5 *μ*m by 0.5–1 *μ*m (Postgate and Campbell 1966). The bacterial morphogenesis and cell size are determined by the two major types of proteins, FtsZ, the tubulin homolog responsible for cell division, and MreB, related to actin, which is involved in cell elongation of rod-shaped bacteria (Marshall et al. 2012).

*D. gigas* genome contains the inhibitor of the FtsZ assembly, the *minCDE* system similar to the one described for *E. coli* (DGI_3156, 3157 and 3158) (Fig. S1A) (de Boer et al. 1989), which is not detected in any other *Desulfovibrio spp* genomes so far sequenced. The Min system was described as participating in the accurate placement of the division site, allowing septum formation in the middle of the cell by inhibiting FtsZ polymerization. In fact, it was shown that the defects in the Min system components lead to a high frequency of aberrant FtsZ assembly at sites immediately adjacent to the cells poles (Rothfield et al. 2005; Marshall et al. 2012).

As *D. gigas* contains the *minCDE* genes, in contrast to other *Desulfovibrio* spp, this may suggest the involvement of the encoded polypeptides in the different size of this bacterium. Indeed, the presence of these genes may originate an inhibition of FtsZ assembly, leading to an increase in cell size. In addition to the Min system, a homolog of the nucleoid occlusion SmlA protein (DGI_2692), that prevents the polymerization of FtsZ and thus cell division, was also found (Bernhardt and de Boer 2005). We further detected a homolog of a third FtsZ assembly inhibitor that was described for *B. subtilis*, the *pgcA* gene (DGI_0235), which couples cell division to cell mass (Weart et al. 2007).

Regarding the MreB, considered as an organizer of cell wall synthesis, three genes encoding similar proteins appear in the *D. gigas* genome (DGI_0336, 0660 and 2254), whereas other *Desulfovibrio* spp. contain only two genes. Although the pathway by which MreB controls the cell width is not yet established, the presence of an extra *mreB* gene could as well contribute to the big size of *D. gigas*. As such, a putative interaction network of *D. gigas* proteins involved in the cell size, built based on the data obtained from *D. vulgaris* protein interactions using the STRING database (http://string-db.org/) and the data available in the literature (Bi and Lutkenhaus 1990; Weart et al. 2007; Fischer-Friedrich et al. 2010; Chien et al. 2012; Hill et al. 2012), can be drawn (Fig. S2).

### Phylogenetic analysis of Desulfovibrio genus

A phylogenetic tree was built based on protein sequences coded by the conserved RpoB and GyrB protein sequences from 21 isolates of *Desulfovibrio* genus whose genomic sequences are available and annotated.

**Figure 2:**
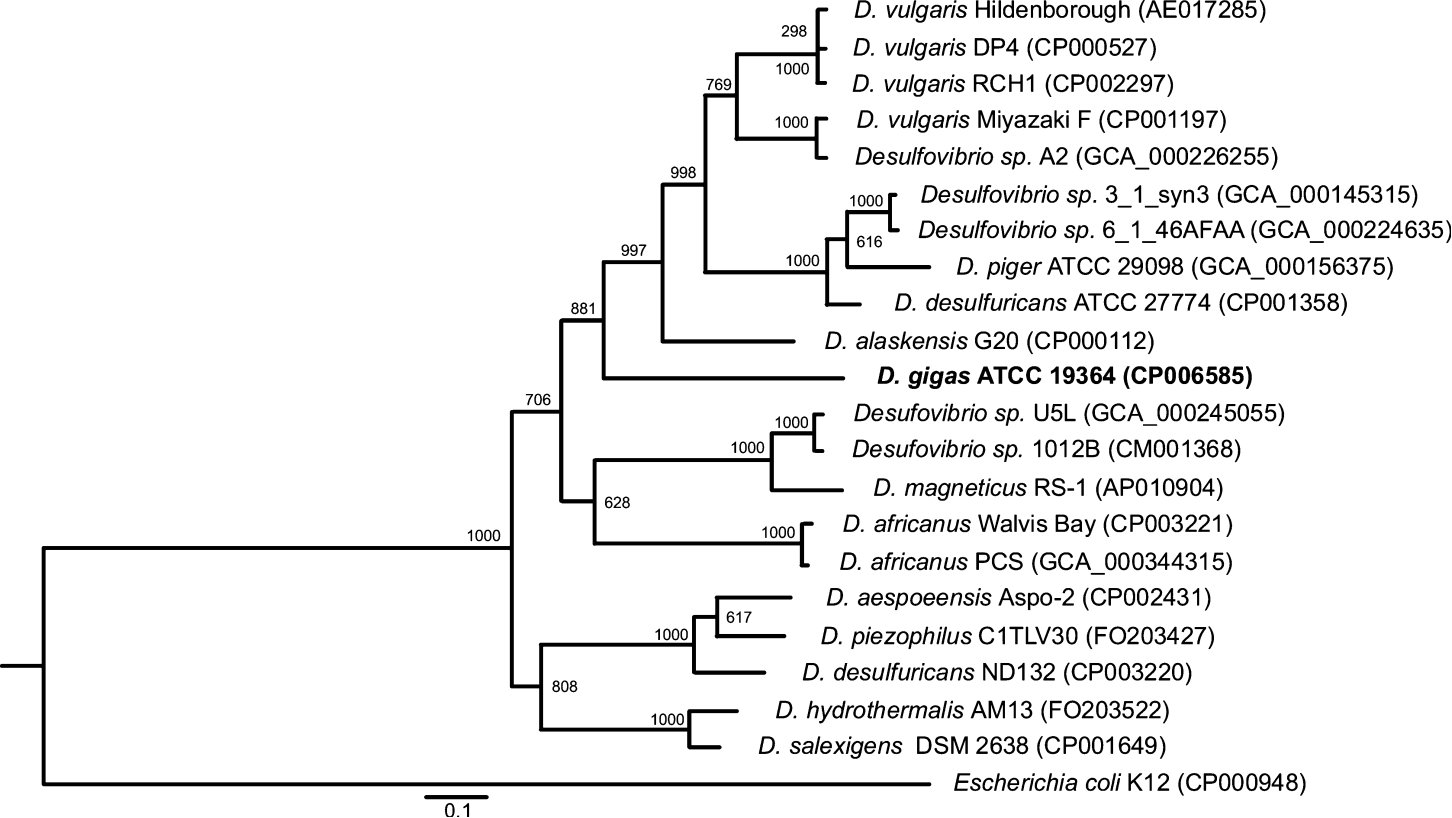
Evolutionary relationship of *Desulfovibrio* species. This tree was built based on RpoB and GyrB protein sequences using a Maximum Likelihood approach with 1000 iterations for the Bootstrap test, both implemented in the PhyML tool. The number at each node corresponds to the frequency of that branching occurred during the 1000 iterations. The sequences of the *E. coli* proteins were applied as outgroup. Accession numbers are indicated after the species names.

The analysis revealed two well-supported deep-branching main clades (Fig.2). Within the upper clade, two groups emerge: one group contains *D. gigas* clustering with *D. alaskensis* G20, *D. piger* ATCC29098, *D. desulfuricans* ATCC27774, and *D. vulgaris* spp; the other group embraces *D. magneticus RS-1*, two *D. africanus* strains, and two not yet assigned *Desulfovibrio* species (Fig.2). The lower clade contains a single group of *Desulfovibrio* species with many of them found in larger depths (piezophilic environment). The tree topology suggests a more divergent evolutionary history of the species included in the lower clade. In fact, gene structures associated with oxygen resistance and detoxification, such as the superoxide dismutase (SOD) genes (DGI_1536 and DGI_3082, Table S7), are present not only in *D. gigas* and in the closely related *D. vulgaris* species, but also in the subgroup containing *D. magneticus* RS-1. However, species observed in the lower clade, such as *D. piezophilus* and *D. hydrothermalis*, do not contain any homologous sequences for SOD genes. This different oxygen resistance gene structures could be the reflex of a different evolutionary process of this later group of *Desulfovibrio* spp since these species are found in environments where O_2_ is present at very low levels (Ji et al. 2013).

Remarkably, according to this phylogenetic analysis, the isolates within *Desulfovibrio* genus not yet classified, namely *Desulfovibrio* sp. 3_1_syn3 together with *Desulfovibrio* sp. 6_1_46AFAA, *Desulfovibrio* sp. U5L along with *Desulfovibrio* sp. 1012B and *Desulfovibrio* sp. A2, are clustered with *D. desulfuricans, D. magneticus, and D. vulgaris*, respectively. Corroborating our data with respect to the *Desulfovibrio* sp. A2, using 16S rRNA gene sequence, a 99.1% overall sequence similarity with *D. vulgaris* Miyazaki was shown (Mancini et al. 2011). These findings indicate that they are closely related species and merit further investigation, in order to clarify their classification within the *Desulfovibrio* genus.

Another interesting aspect of this analysis relies in the positioning of *D. desulfuricans* ND132 within the lower clade of the phylogenetic tree, rather than in the upper clade, where *D. desulfuricans* appears (Fig.2). This finding has already been observed by others and strongly indicates that its classification should be reconsidered (Brown et al. 2011; Gilmour et al. 2011).

### CRISPR/Cas systems in the *D. gigas* genome

CRISPRs are loci encompassing several short repeats functioning as an adaptive microbial immune system, that have also been shown to limit horizontal gene transfer (HGT) by preventing conjugation and plasmid transformation (Marraffini and Sontheimer 2008). Several types of CRISPR-associated proteins (Cas) are encoded by *cas* genes located in the vicinity of CRISPRs. Cas proteins are required for the multistep defense against intruder genetic elements. Their number, identity, and the corresponding operon organization appear to be extremely variable. Makarova et al. (2011) have proposed a classification of CRISPR/Cas systems in which the *cas1* and *cas2* genes constitute the core of three distinct types of system. Each system was further divided into different subtypes, on the basis of the gene composition and architecture of the respective operons.

**Figure 3:**
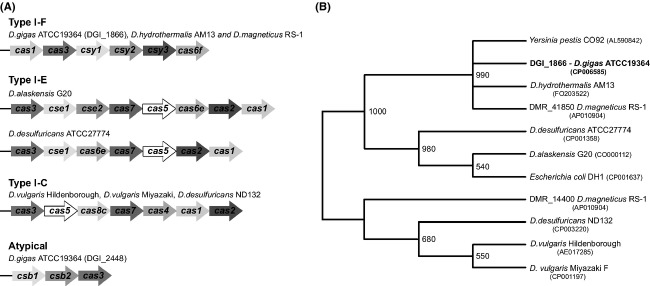
Distribution of different types of CRISPR/Cas systems among *Desulfovibrio* spp. (A) Operon structure of *cas* genes from the indicated *Desulfovibrio* spp. The operon organization was assessed using the DOE Joint Genome Institute (JGI) website (http://www.jgi.doe.gov/). Classification into the distinct Type I subtypes is according to (Makarova et al. 2011). (B) The evolutionary history of Cas1 proteins was inferred by using the Maximum Likelihood method. The bootstrap consensus tree inferred from 1000 replicates was taken to represent the evolutionary history of the taxa analyzed. Branches corresponding to partitions reproduced in less than 50% bootstrap replicates were collapsed. The percentage of replicate trees in which the associated taxa clustered together in the bootstrap test (1000 replicates) is shown next to the branches. Accession numbers are indicated after species name.

In the particular case of *Desulfovibrio* spp., little is known about the presence of CRISPR sequences and Cas-associated genes. *D. vulgaris* Hildenborough appears to have a plasmidic CRISPR/Cas locus that falls into the subtype I-C system, according to the above mentioned classification criteria (see Fig.3A and Makarova et al. (2011), Haft et al. (2005)). A survey of the genome of *D. gigas* for CRISPR repeats, revealed the presence of 6 CRISPR repeats with two of them being flanked by Cas operons (Table S5 and Fig3A). One of the *D. gigas* CRISPR/Cas systems fall into the I-F type, for the first time reported in *Yersinia pestis*, and the other one does not fit in any of the known types of CRISPR/Cas systems (Fig.3A).

Using a dedicated database (http://crispi.genouest.org/) (Rousseau et al. 2009), we searched for CRISPR sequences that have adjacent *cas* genes among the different species of *Desulfovibrio* genus. We have focused on CRISPR/Cas arrays that possess the ubiquitous core protein Cas1, which is involved in new spacer acquisition. We then used the conserved Cas1 protein as a scaffold to investigate the evolution of the CRISPR/Cas system in the *Desulfovibrio* genus (Fig.3B). Remarkably, CRISP/Cas systems are absent from the genome of *D. aespoeensis* Aspo-2, *D. africanus* Walvis Bay, *D. piezophilus* C1TLV30, and *D. salexigens* DSM2638.

The phylogenetic tree of *Desulfovibrio* genus was used in order to explore the evolutionary bases of the CRISPR/cas loci (Fig2). In the particular case of group I, the topology of Cas1 phylogenetic tree (Fig.3B) together with the RpoB and GyrB based phylogeny of the genus *Desulfovibrio* (Fig2), strongly suggests the divergence after speciation of an ancestor gene common to *D. gigas* ATCC19364, *D. hydrotermalis* AM13, and *D. magneticus* RS-1. Furthermore, the Cas1 phylogeny shows *D. desulfuricans* ATCC27774 and *D. alaskensis* G20 grouping separately from the other *Desulfovibrio* spp. and of *E. coli* DH1 (Fig.3B). These phylogenetic relationships together with the RpoB_GyrB phylogenetic tree indicate that CRISPR/Cas system I-E (group II) might have been acquired from HGT during prokaryotic evolution. Indeed, a comprehensive phylogenetic analysis of CRISPR/cas loci points toward their propagation via HGT events (Godde and Bickerton 2006). Regarding group III, it seems that the CRISPR/Cas subtype I-C is scattered across several *Desulfovibrio* spp. (Figs.2, 3B). The absence of additional *Desulfovibrio* orthologues suggests that the acquisition of this CRISPR/Cas subtype may rely as well in HGT occurrences throughout evolution. Notably, *D. vulgaris* Hildenborough contains the CRISPR/cas locus in its megaplasmid, whereas the closely related *D. vulgaris* Miyazaki (Fig.2) possesses a similar CRISPR/cas array in the chromosome. Godde and Bickerton have proposed that most megaplasmids should not be stably maintained in their host cells (Godde and Bickerton 2006). Consistently, the lack of a megaplasmid in *D. vulgaris* Miyazaki indicates that a recent HGT event might have been responsible for the appearance of CRISPR locus in *D. vulgaris* Hildenborough.

### Strategies to survive oxygen and nitric oxide

SRB, in the diverse environmental niches they occupy, can come across with reactive oxygen or nitrogen species that cause oxidative damage to the cells. Formerly classified as strict anaerobes there is, however, growing evidence that they are able to cope with oxygen and to use it to produce ATP even if they are unable to grow in its presence. As such, the organisms have developed several strategies to avoid such damage.

**Figure 4:**
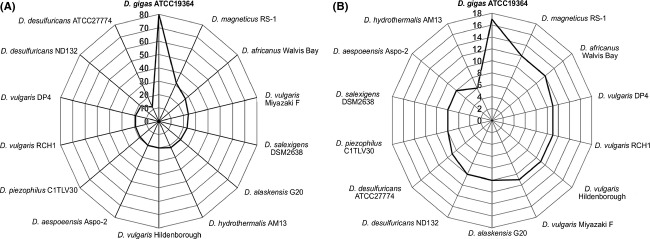
Radar Graphs comparing orthologs of *D. gigas* within the genome of sequenced *Desulfovibrio* spp. (A) Genes involved in chemotaxis response; (B) genes involved in O_2_ sensing; Orthologs search was conducted using the EDGAR database.

The response to different oxygen concentrations in microorganisms, aerotaxis, is often initiated by the transmembrane chemoreceptors, the methyl-accepting chemotaxis proteins, and involves many other proteins organized in a cascade of reactions activating the flagellar motor, allowing the cells to move to an optimal oxygen gradient (Armitage 1997). SRB within the microbial mats and oxic environments are motile, and active movements are observed in response to change in oxygen gradients which were interpreted as a strategy to survive in these environments (Krekeler et al. 1989; Canfield and Des Marais 1991; Teske et al. 1998; Eschemann et al. 1999). The sensing of extra and/or intracellular signals is followed by their transduction to the transcriptional and post-transcriptional machineries. As it was previously demonstrated, *D. gigas* contains an operon encoding the chemotaxis proteins CheB, CheR, CheW, CheY, and CheA, that are co-transcribed as an 11 kb mRNA whose expression is not altered either by O_2_ or nitric oxide (NO, Felix et al. 2006). By searching *D. gigas* genome, many other chemotaxis coding regions were found scattered throughout the genome (Table S6). A comparison of the newly identified genes coding for chemotaxis proteins against other sequenced *Desulfovibrio* spp., indicate that few of these operons have orthologous in closely related species such as *D. vulgaris* or *D. desulfuricans* strains (Fig.4A). As such, it is clear that the genes without orthologs represent specific mechanisms that *D. gigas* uses to sense and avoid unfavorable aerobic conditions. Strikingly, the closely related *D. vulgaris* DP4 and RCH1 as well as *D. desulfuricans* are those among the *Desulfovibrio* spp. that have fewer orthologs genes encoding chemotaxis polypeptides when compared to *D. gigas* (See Fig.4A).

Besides sensing, these microorganisms have developed a network of defense mechanisms against reactive oxygen species (ROS), being the toxic O_2_ eliminated by dismutation to H_2_O_2_ and O_2_, a reaction catalyzed by the SOD (dos Santos et al. 2000). The accumulation of toxic H_2_O_2_ is further eliminated by the catalase which is found in *D. gigas* genome as a single gene (DGI_2858) (dos Santos et al. 2000). *D. gigas* contains in its genome two SOD genes, one named neelaredoxin and another one (DGI_1536) here described for the first time (see Table S7). Neelaredoxin from *D. gigas* was shown to be a bifunctional protein that has both superoxide reductase and SOD activities. (Silva et al. 1999; Abreu et al. 2002). *D. gigas* genome also contains genes encoding three rubrerythrins, one peroxiredoxin, one rubredoxin-like protein, and three F390 synthetase proteins (Table S7), which have been shown to be related to defense mechanisms against oxidative stress.

As illustrated in the radar chart, 17 genes are involved in O_2_ metabolism of *D. gigas* some of which have orthologs in other species of *Desulfovibrio*. As such*, D. gigas* shares 12 genes with *D. magneticus* RS-1 and 6 genes with *D. hydrothermalis* AM13, a more distant species (Fig.4B). Interestingly, when observed in more detail, the species grouped together with *D. hydrothermalis* AM-13 in the phylogenetic analysis (Fig.2), such as *D. aespoeensis* and *D. salexigens* showed an increased number of superoxide reductases (two genes) when compared to *D. gigas* or *D. vulgaris*, that only possesses one gene, according to the SORGOdb database (Lucchetti-Miganeh et al. 2011).

**Figure 5:**
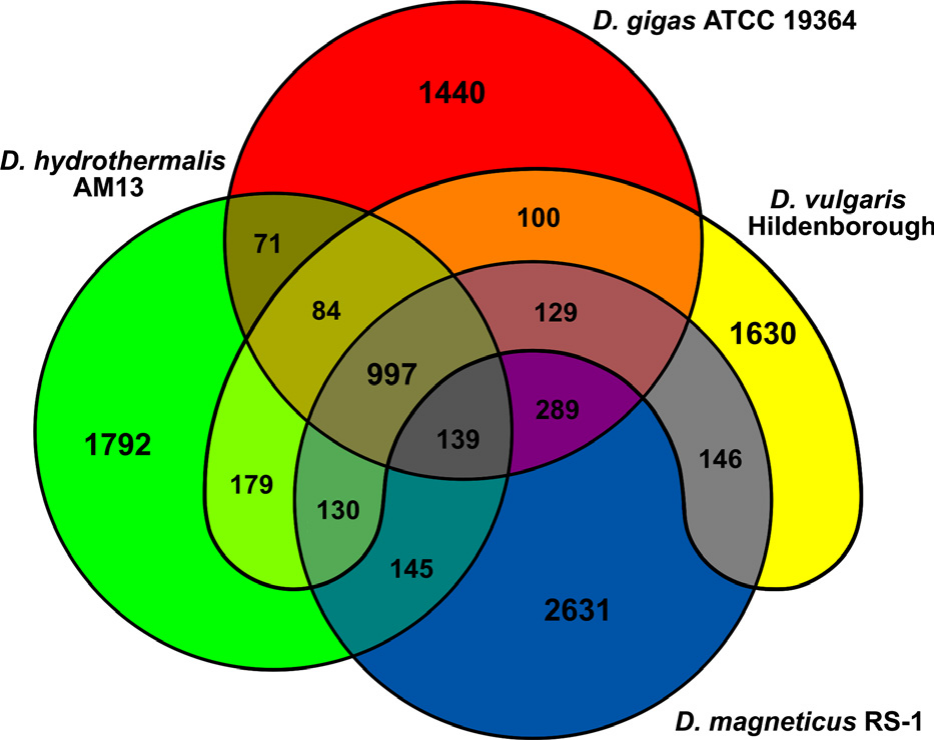
Protein orthology comparison among genomes of *Desulfovibrio gigas, D. magneticus* RS-1*, D. hydrothermalis* AM 13, and *D. vulgaris* Hildenborough. The Venn diagram shows shared ortholog groups for any given gene of each species under analysis.

Furthermore, it is also interesting to notice that *D. gigas* presents a higher number of orthologous proteins regarding O_2_ sensing and detoxification with *D. magneticus* RS-1 and *D. africanus* Walvis Bays species than with the more closely related *D. alaskensis* G20 and *D. vulgaris* (Fig.4A and B). A whole-genome orthology analysis using the EDGAR database confirms this fact, as in general, a higher number of orthologous groups are observed between *D. gigas* and *D. magneticus* RS-1 than with *D. vulgaris* Hildenborough (Fig5). This result was not expected on the basis of the phylogenetic results obtained, since *D.gigas* is more closely related to *D.vulgaris* Hildenborough than to *D.magneticus* RS-1. It is possible that only specific groups of genes included in the category of chemotaxis and detoxification show similarity to *D. magneticus* RS-1.

Another mechanism of O_2_ detoxification involves the participation of the flavodiiron protein, rubredoxin:oxygen reductase (ROO) (Chen et al. 1993), which was also shown to protect *D. gigas* against nitrosative stress by acting as a NO reductase (Rodrigues et al. 2006b). Under nitrosative stress, *roo* transcription is regulated by NorR (NorR1L). A second putative *norR* gene designated as *norR2L* was found in *D. gigas* genome (Table S8) although its function is still unknown (Varela-Raposo et al. 2013). *D. gigas* genome also includes one copy of ‘hybrid cluster protein’ (HCP), a protein with an unusual structure (Cooper et al. 2000) proposed to have a function in nitrogen cycle due to its hydroxylamine reductase activity (Wolfe et al. 2002; Cabello et al. 2004; Overeijnder et al. 2009). A role in defense against oxidative stress has also been suggested for HCP on the basis of its peroxidase activity (Almeida et al. 2006). While in other *Desulfovibrio* spp., HCP is co-expressed with a hypothetical ferredoxin (*frdx*) gene (Rodionov et al. 2004) in *D. gigas* it is encoded by a monocistronic gene (Fig. S1B). It is also important to mention that *hcpR*, a gene encoding a transcriptional regulator of *hcp* expression identified in other *Desulfovibrio* spp., was also observed in *D. gigas* upstream of *hcp* although localized in opposite direction (Table S9, Fig. S1B) (Cadby et al. 2011). *D. gigas* genome encodes also the membrane complex cytochrome *c* nitrite reductase (NrfHA), which is suggested to play a role in nitrite detoxification since no growth on nitrite or nitrate is reported for *D. gigas*, as well as for *D. vulgaris*. (Greene et al. 2003; He et al. 2006). Other nitrate reductases as well as nitroreductases encoded in *D. gigas* genome (Table S8) might be involved in NO detoxification mechanisms.

### Central carbon metabolism

*D. gigas* accumulates large amounts of polyglucose as an endogenous carbon and energy reserve, utilizing these sugar compounds for growth (Fareleira et al. 1997). We have conducted a broad analysis in its genome to identify the elements of the central carbon metabolism involved in many different pathways (Table S11 to S17). Biochemical studies have shown (Fareleira et al. 1997), that *D. gigas* contains all the genes encoding proteins of the Embden-Meyerhof pathway (Table S13), whereas the genes coding for the hexokinase and the 2-keto-3-deoxygluconate 6-phosphate (KDGP) aldolase of the Entner-Doudoroff pathway are lacking (Table S14). *D. gigas* belongs to SRB group of incomplete-oxidizers, producing acetate and CO_2_ as its main end-products from substrate oxidation. Inspection of the genome reveals that the genes corresponding to 2-oxoglutarate dehydrogenase, 2-oxoglutarate synthases, and both subunits of the succinyl Co-A ligase, *sucC* and *sucD* (Table S15) from the tricarboxylic acid (TCA) cycle are absent. Both copies of the succinate:quinone oxidoreductase (SQR), one of each is identified here (DGI_0826 to DGI_0828 - Table S16), appear to function mainly as fumarate reductases rather than as succinate dehydrogenases, due to a conserved glutamine residue (Glu180) in the Sdh/FdrC subunit (Zaunmuller et al. 2006).These results indicate that both oxidative and reductive TCA cycle pathways are not fully functional and are likely to have a biosynthetic function, as suggested for *D.vulgaris* Hildenborough (Heidelberg et al. 2004). In the Wood-Ljungdahl pathway (Table S17) the genes coding for a key element from this pathway (Ragsdale and Pierce 2008), the bifunctional carbon monoxide dehydrogenase/acetyl-CoA synthase (CODH/ACS) enzyme, are absent in *D. gigas*. Instead, like some *Desulfovibrio* spp. such as *D. magneticus* and *D. africanus* strains, *D. gigas* genome codes for an aerobic-type CODH of the *coxSLM* type, similar to the CO dehydrogenase of *Oligotropha carboxidovorans* (Dobbek et al. 1999). This enzyme shows a high sequence similarity with the aldehyde oxidoreductase (MOP) from *D. gigas* itself (Romao et al. 1995). This may suggest that this CO dehydrogenase could play a function in oxygen metabolism and resistance in *D. gigas* rather than being part of the Wood-Ljungdahl pathway as is the case of *D. vulgaris* Hildenborough, which presents a *codh/acs* gene. Furthermore, the absence of the bifunctional enzyme in *D. gigas* indicates that in contrast to *D. vulgaris* Hildenborough, CO cycling (Voordouw 2002) is not an effective mechanism of energy conservation.

### Energy metabolism

A survey of *D. gigas* genome revealed several genes encoding dehydrogenases that oxidize organic acids and alcohols, as well as putative transporters and permeases for these substrates (Tables S18 to S20). Pyruvate, the main metabolic intermediate of organic carbon oxidation can be oxidized by the two pyruvate oxidoreductases (DGI_0996 and DGI_1712/DGI_1713) as well as by other oxo-organic acid ferredoxin: oxidoreductases enzymes present (Table S21). Although *D. gigas* genome reveals many genes encoding such complexes, the pyruvate:formate lyase (*pfl*), a gene involved in fermentative metabolism, was not identified. This enzyme produces acetyl-CoA and formate when pyruvate is the main carbon and energy source. As suggested for *D. vulgaris* Hildenborough, formate cycling could contribute to energy conservation in a mechanism similar to CO or hydrogen cycling (Voordouw 2002; Heidelberg et al. 2004). The apparent absence of this gene in *D. gigas* suggests that formate cycling is not occurring although this bacterium is able to grow using formate as the main electron donor (our unpublished results), since it presents two genes encoding formate dehydrogenases (Table S20). One of these enzymes, a tungsten seleno-protein, was already described (Almendra et al. 1999), whereas the second has not been reported to our knowledge (DGI_3334 and DGI_3335).

As other *Desulfovibrio* spp*., D. gigas* grows chemolithotrophically deriving energy from hydrogen oxidized in the periplasm by hydrogenases, coupled to sulfate reduction in the cytoplasm, creating a proton gradient ultimately used to generate ATP through F_1_F_0_-ATP synthase (Table S22). The electrons generated in the periplasm, by periplasmic hydrogenase activity, are transferred through the membrane for the sulfate reduction, in the cytoplasm, by multiheme c_3_-type cytochromes (at the periplasmic side) and membrane-bound electron transport complexes.

The presence of at least three *c*_*3*_-type cytochromes was found in *D. gigas* genome (Table S23). The full set of genes necessary for the dissimilatory sulfate reduction to sulfide were also detected, as well as specific sulfate permeases (Table S10). Interestingly enough, in the case of the ATP-synthase, not only the genes encoding the F_1_F_0_-ATP synthase were identified (Table S22) but another ATP-synthase, which apparently is not present in other *Desulfovibrio* spp, was found (Fig. S1A). This enzyme is similar to the Vacuolar-type ATPases (V_o_V_1_) and in some anaerobic bacteria, such as *Enterococcus hirae*, it functions as a sodium pump (Kakinuma et al. 1999). In *D. gigas,* this second ATPase could enhance ATP production derived from transmembrane electrochemical proton gradient.

In contrast to other *Desulfovibrio* spp. genomes so far sequenced (Pereira et al. 2011), only two [NiFe] type hydrogenase are present in *D. gigas*: the periplasmic HynAB (Volbeda et al. 1995) and the energy conserving Ech hydrogenase (Rodrigues et al. 2003) (Table S24). Recent work performed using mutant strains for these genes indicates that, although it is possible that the hydrogen cycling model of energy conservation (Odom et al. 1981) is effective, it appears to contribute substantially less to the final energy yield *of D. gigas* as proposed for other *Desulfovibrio* spp. (Morais-Silva et al. 2013). This could be a reflex of the unusual low number of these enzymes in *D. gigas*. An attempt to generate a double mutant strain in both hydrogenases (unpublished data) indicated that at least one of these hydrogenases might be essential for cell viability. Indeed, the double mutant was unable to grow in diverse respiratory and fermentative conditions.

### Energy conservation

**Figure 6:**
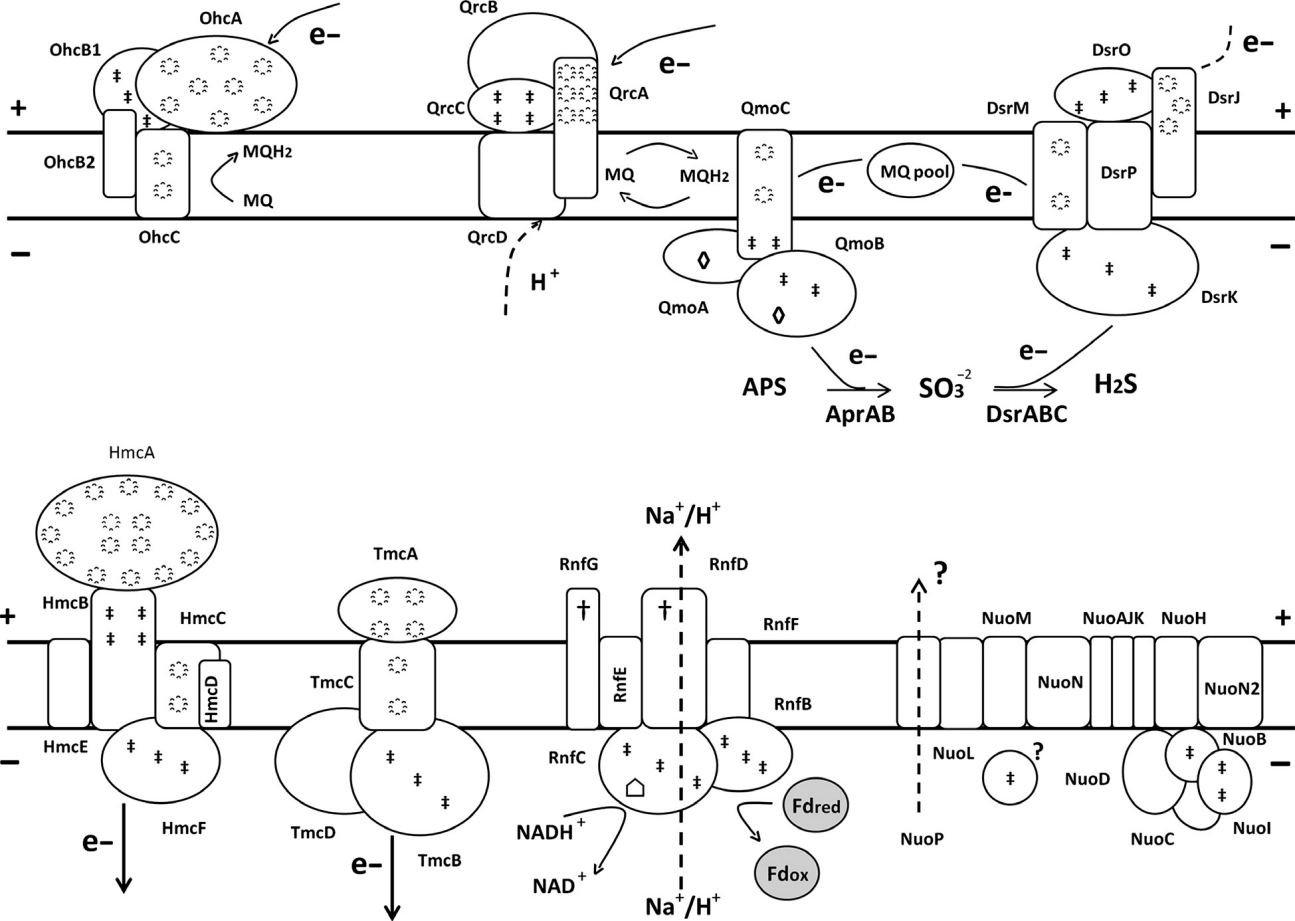
Schematic representation of membrane-bound electron-transfer complexes present in *D. gigas* genome. The complexes were identified in the genome according to their predicted function*:* quinone reduction, Ohc and Qrc; quinol oxidation, Qmo; transmembrane electron transfer/sulfite reduction DsrMKJOP, Hmc and Tmc; and NADH/Fd oxidation, Rnf and Nuo. Symbols represent:: 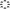, heme; ‡, iron sulfur center; †, FMN cofactor; ⌂, flavin cofactor. and ◊, FAD cofactor. Dashed lines represent hypothetical pathways for electron/proton flow.

Sulfate reducers contain several transmembrane redox complexes involved in energy metabolism and conservation (Pereira et al. 2011) (Fig6 and Table S25). The genome of *D. gigas* encodes two transmembrane multiheme cytochrome *c* complexes, Tmc and Hmc, described as participating in electron transfer from periplasmic hydrogen oxidation to sulfite reduction as transmembrane electron circuits (Rossi et al. 1993; Pereira et al. 2006). An octa-haem cytochrome c complex (Ohc), proposed to transfer electrons from the periplasm to the quinone pool, due to the absence of the cytoplasmic CCG protein, was also observed. Furthermore, we identified the quinone interacting membrane-bound oxidoreductase complex (*qmoABC*) and the transmembrane electron transfer DsrMKJOP complex, both related to sulfate reduction and suggested to act in the electron transfer to the final reductases, Apr (*aprAB*) and Dsr (*dsrABC*), respectively (Pires et al. 2003; Dahl et al. 2005). The presence of the Qrc (*qrcABCD*) quinone reduction complex, which was shown to transfer electrons from the Tpl-*c*_*3*_ cytochrome to the menaquinone during sulfate respiration in a quinone:menaquinone loop together with the Qmo complex (Venceslau et al. 2010), suggests the existence in *D. gigas* of a mechanism of energy conservation linking periplasmic hydrogen or formate oxidation to cytoplasmic sulfate reduction. In addition, complexes involved in NAD(P)H and ferredoxin oxidation were identified (Table S25). An operon coding for the NADH:quinone oxidoreductase (*nuo*), firstly reported in *D. magneticus* RS-1 (Nakazawa et al. 2009) was also detected. This enzyme complex is proposed to couple NADH oxidation to proton translocation (Spring et al. 2012). However, the genes encoding the NADH dehydrogenase module (*nuoEFG*) are absent, suggesting a different electron donor, such as ferredoxin (Fd), instead of NADH (Pereira et al. 2011). Notably, a complex with high similarity to the *nuo* complex, the Mnh Na+/H+ antiporter, that was not detected in other *Desulfovibrio* spp. genomes, is present in *D. gigas* (Fig. S1A). This complex is suggested to function as a transmembrane electron potential-generating NADH dehydrogenase rather than as a secondary transmembrane electron potential-consuming antiporter, directly accounting for the great transmembrane electron potential in *Staphylococcus aureus* (Bayer et al. 2006). The presence of a similar mechanism in *D. gigas* might compensate for the apparent lack of energy conservation through metabolite cycling mechanisms, such as CO, formate or hydrogen cycling, deduced from its genome.

**Figure 7:**
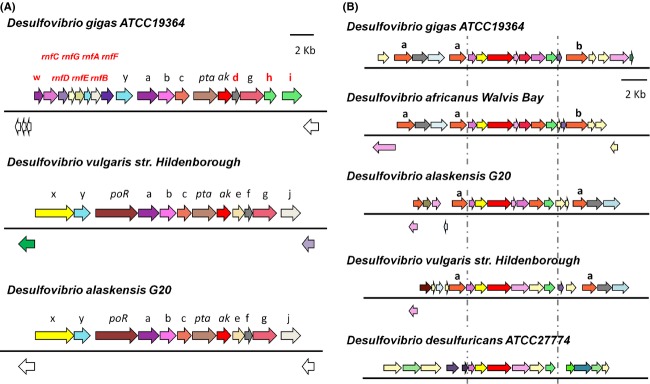
Genomic organization of the operons involved in the energy metabolism of *D.gigas*. (A) Comparison of the *Desulfovibrio spp* genomic regions containing the D-Lactate dehydrogenase operon. Differences in genomic organization are with red letters and genes are indicated as follows: *poR* - Pyruvate-ferredoxin oxidoreductas; *pta -* phosphate acetyltransferase; *ak* - acetate kinase; *rnfC, rnfD, rnfE, rnfG, rnfA, rnfB, rnfF -* Rnf complex; *w* - cytochrome c type protein; *x* - response regulator; *y* - sigma-54 response regulator; *a*, *b* and *c* - cytochrome c, lactate permease, and iron sulfur cluster protein (Ldh1a) subunits of the D-lactate dehydrogenase,; *d* - lactate utilization protein B/C; e and f - hypothetical proteins; *g* - iron sulfur cluster binding protein (Ldh1b); *h* - FMN-dependent *α*-hydroxyacid dehydrogenase; *i -* Sodium-dependent transporter; *j -* methyl-accepting chemotaxis protein. Identification of the single letter genes was made according to BLAST best hit value. Gene cluster organization of *D.vulgaris* Hildenborough and *D.alaskensis* G20 were obtained at the DOE Joint Genome Institute: http://www.jgi.doe.gov/. (B) Organization of genomic regions containing the HdrABC/FloxABCD operon and neighboring genes. Hdr/Flox operon appears between dashed lines and is composed of three subunits of Hdr-like proteins and four subunits of the flavin oxidoreductase genes (Flox) in *D.gigas*, *D.africanus,* and *D.alaskensis* or three subunits in *D.vulgaris* Hindelborough and *D.desulfuricans* ATCC27774. *α-*alcohol dehydrogenase; *β*-aldehyde dehydrogenase.

A search of the *D. gigas* genome also revealed the presence of the Rnf complex (*rnfCDGEABF*), proposed to function as a Na^+^-translocating, ferredoxin:NAD^+^ oxidoreductase (Biegel and Muller 2010) and a multiheme cytochrome *c* in the same operon (Fig7A–w), hypothesized to mediate the electron transfer between the periplasmic cytochrome *c* pool and the cytoplasmic NAD(P)H/Fd (Li et al. 2006; Pereira et al. 2011). Another gene with similarity to cytochrome *c* is found adjacent to the Rnf complex in *D. gigas*, corresponding to cytochrome c subunit of D-lactate dehydrogenase (Fig.7A– a). Interestingly, the *rnf* operon is not present in the genomic context of this dehydrogenase in other *Desulfovibrio spp*., being replaced by the pyruvate:oxidoreductase (*poR*). This fact may indicate that the Rnf complex in *D. gigas* could be directly involved in the electron transport from lactate to Fd/NADH or between these two elements.

Another group of energy-conserving enzymes and complexes are those related to electron bifurcation processes. *D. gigas* genome encodes two paralogous (Table S26) heterodimeric transhydrogenase (NfnAB), responsible for the reversible NADH-dependent reduction of NADP^+^ by Fd (Wang et al. 2010).

Only one cytoplasmic hydrogenase was observed in *D. gigas* genome. We have, however observed a sequence of an electron bifurcating complex: the HdrABC/FloxABCD (Fig7B). Flox gene products are likely to oxidize NAD(P)H and transfer electrons to the HdrABC proteins (Pereira et al. 2011) (Table S27). These genes are found in other *Desulfovibrio* spp., such as *D. vulgaris* and *D. alaskensis*, between two alcohol dehydrogenases (Fig7B– a), suggesting that they might be involved in the electron transfer from alcohol substrates. The presence of an aldehyde dehydrogenase (Fig.7B– b), found downstream of this operon in *D. gigas*, as well as *D. africanus* Walvis Bay, might indicate that this complex could also use aldehydes as another electron source to this complex. This genomic arrangement suggests that not only alcohol but also aldehyde oxidation could participate in mechanisms of energy conservation in *D. gigas*.

## Conclusions

The observations reported for the genome of *D. gigas* ATCC19364 highlight the differences found within several species of the *Desulfovibrio* genus. The larger size of *D. gigas* cells when compared to other *Desulfovibrio* spp. might be a reflex of the presence of FtsZ inhibitors, such as the MinCDE system, which was not described for any members of this genus. In accordance, the presence of a single rRNA operon and multiple CRISPR/Cas elements specific for this species might be involved in the phylogenetic separation of *D. gigas*, placing it more closely related to *D. vulgaris* and *D. desulfuricans* strains. However, the presence of a different composition of genes involved in certain metabolic aspects, like sensing and response to oxygen and NO stress, highlighted by the presence of a new SOD, a second *norR* transcriptional factor (NorRL2), several putative nitrate reductases and an aerobic-type CODH, reveal a greater number of orthologous groups with more distant related species like *D. magneticus*. This also indicates a highly developed and flexible enzymatic machinery to overcome the deleterious effects of an aerobic environment. This flexibility can be further detected in the genes involved in the energy metabolism and conservation, as new proteins (Fdr and Fdh) and complexes, such as a secondary vacuolar-type ATPase and two complexes linking NAD(P)H and ferredoxins with electron transfer (Nuo and Mnh) were identified. On the other hand, a low number of hydrogenases and the absence of *codh/acs* and *pfl genes* indicate that the intermediate compounds (H_2_, CO, and formate) do not contribute to mechanisms of energy conservation in *D. gigas* as much as they do in other *Desulfovibrio* spp. Despite that, recent experimental analysis performed using mutants for genes encoding hydrogenases demonstrates that at least one hydrogenase is required for cell viability. Interestingly, specific genomic elements, like the presence of a cytochrome *c* in the Rnf complex and an aldehyde dehydrogenases in the vicinity of the Hdr/Flox operon may provide alternative routes for energy conservation processes, that could compensate the absence of the above mentioned genes or multiple hydrogenases. This might indicate that different substrates (alcohols and aldehydes) and coenzymes (NAD^+^/NADP^+^) could play a more important role in redox reactions of *D. gigas* than previously thought.

## Supporting Information

Figure S1 Specific genomic organization of *Desulfovibrio gigas*. (A) Organization of the *hcp* and *hcpR* monocistronic operons in *D.gigas* in comparison with other *Desulfovibrio* species, where: *frdx*, ferredoxin; *a*, Upsa-like protein; *b*, alcohol dehydrogenase; *c*, sensory box histidine kinase; *d*, acpD:acyl carrier protein phosphodiesterase; *e*, putative lipoprotein; *f*, polysaccharide export protein; *g*, cupin 2 conserved barrel domain protein. Gene cluster organization from *D. vulgaris* Hildenborough, *D. alaskensis* G20, and *D. desulfuricans* ATCC27774 were obtained at the DOE Joint Genome Institute (http://www.jgi.doe.gov/). (B) Organization of operons exclusively present in *D. gigas* genome as compared to other *Desulfovibrio* spp.: (i) aerobic-type carbon monoxide dehydrogenase complex; (ii) vacuolar-type ATP-synthase complex; and (iii) multisubunit Na^+^/H^+^ antiporter complex. Genes were assigned according to the predicted protein function. The unnamed coding regions are either hypothetical proteins or proteins of unknown function.

Figure S2 Interaction network of *Desulfovibrio gigas* proteins involved in cell size. Purple circles indicate central elements of the network. Yellow circles indicate elements with a fewer number of interactions. Blue lines show protein interactions common to several *D*. genus as retrieved by the STRING database,whereas red lines correspond to *D. gigas* specific interactions.

Table S1 COG functional groups. **Table S2.** Codon usage. **Table S3.** Transposable elements. **Table S4.** Selenocystein-containing proteins. **Table S5.** CRISPR proteins. **Table S6.** Chemotaxis proteins. **Table S7.** Response to oxygen. **Table S8.** Nitrogen metabolism. **Table S9.** Transcriptional factors sigma 54. **Table S10.** Sulfate metabolism. **Table S11.** Pentose phosphate pathway. **Table S12.** Beta oxidation. **Table S13.** Embden-Meyerhof-Parnas pathway. **Table S14:** Entner-Doudoroff pathway. **Table S15.** TCA cycle. **Table S16.** Fumarate metabolism. **Table S17.** WoodLjungahl pathway. **Table S18.** Alcohol metabolism. **Table S19.** Lactate metabolism. **Table S20.** Formate metabolism. **Table S21.** Oxidation of pyruvate to acetyl-CoA and acetate formation. **Table S22.** ATP synthesis. **Table S23.** Cytochromes. **Table S24.** Hydrogenases. **Table S25.** Membranar energy complexes. **Table S26.** Nfn complexes. **Table S27.** Hdr-like proteins.
