# Supplementary material for: Genome sequence of the model sulfate reducer Desulfovibrio gigas: a comparative analysis within the Desulfovibrio genus
Source: Microbiologyopen. 2014 Jul 23;3(4):513–30. doi: 10.1002/mbo3.184 (PMC4287179; doi:10.1002/mbo3.184)
Supplement: Figure S1 — Specific genomic organization of Desulfovibrio gigas. (A) Organization of the hcp and hcpR monocistronic operons in D.gigas in comparison with other Desulfovibrio species, where: frdx, ferredoxin; a, Upsa-like protein; b, alcohol dehydrogenase; c, sensory box histidine kinase; d, acpD:acyl carrier protein phosphodiesterase; e, putative lipoprotein; f, polysaccharide export protein; g, cupin 2 conserved barrel domain protein. Gene cluster organization from D. vulgaris Hildenborough, D. alaskensis G20, and D. desulfuricans ATCC27774 were obtained at the DOE Joint Genome Institute (http://www.jgi.doe.gov/). (B) Organization of operons exclusively present in D. gigas genome as compared to other Desulfovibrio spp.: (i) aerobic-type carbon monoxide dehydrogenase complex; (ii) vacuolar-type ATP-synthase complex; and (iii) multisubunit Na+/H+ antiporter complex. Genes were assigned according to the predicted protein function. The unnamed coding regions are either hypothetical proteins or proteins of unknown function. [file mbo30003-0513-sd1.docx]

**SUPPORTING INFORMATION**

**FIGURES**

**ORGANIZATION OF OPERONS**……………………………………………….……..……………… Figure S1

**INTERACTION NETWORK OF PROTEINS INVOLVED IN CELL SIZE**………………………… Figure S2

**TABLES**

**GENERAL GENOME INFORMATION**

COG functional groups…………………………………………………………………… Table S1

Codon usage .………….………………………………………………………………… Table S2

**MISCELLANEOUS**

Transposable elements ………………………………………………………………….. Table S3

Selenocystein-containing proteins ……………………………………………………… Table S4

CRISPR proteins …………………………………………………………………………. Table S5

Chemotaxis proteins ……………………………………………………….…………….. Table S6

**GENERAL METABOLISM**

Response to Oxygen ……………………………………………………………………… Table S7

Nitrogen Metabolism………………………………………………………………………. Table S8

Transcriptional Factors Sigma 54………………………………………………………... Table S9

Sulfate Metabolism ……………………………………………………………………..…. Table S10

**CENTRAL AND ENERGY METABOLISM**

Pentose phosphate Pathway …………………………………………………………….. Table S11

Beta oxidation …………………………………………………………………….……….. Table S12

Embden-Meyerhof-Parnas Pathway………………………………………………….…. Table S13

Entner-Doudoroff Pathway…………………………………………………………….…. Table S14

TCA Cycle………………………………………………………………………………….. Table S15

Fumarate Metabolism……………………………………………………………………… Table S16

WoodLjungahl Pathway…………………………………………………………………... Table S17

Alcohol metabolism ……………………………………………………………………….. Table S18

Lactate metabolism………………………………………………………………………... Table S19

Formate Metabolism……………………………………………………………………..... Table S20

Oxidation of pyruvate to acetyl-CoA and acetate formation………………………….. Table S21

ATP synthesis ……………………………………………………………………………… Table S22

Cytochromes……………………………………………………………………………….. Table S23

Hydrogenases ……………………………………………………………………………… Table S24

**ENERGY CONSERVATION**

Membranar energy complexes ...………………………………………………………… Table S25

Nfn complexes ……………………………………….……………………………………. Table S26

Hdr-like proteins …………………………………………………………………………… Table S27

**Legends to Figures**

**Figure S1.** Specific genomic organization of *D.gigas*. **(A)** Organization of the *hcp* and *hcpR* monocistronic operons in *D.gigas* in comparison with other *Desulfovibrio* species, where: *frdx*, ferredoxin; *a*, Upsa-like protein; *b*, alcohol dehydrogenase; *c*, sensory box histidine kinase; *d*, acpD:acyl carrier protein phosphodiesterase; *e*, putative lipoprotein; *f*, polysaccharide export protein; *g*, cupin 2 conserved barrel domain protein. Gene cluster organization from *D.vulgaris* Hildenborough, *D.alaskensis* G20 and *D. desulfuricans* ATCC27774 were obtained at the DOE Joint Genome Institute (<http://www.jgi.doe.gov/>). **(B)** Organization of operons exclusively present in *D.gigas* genome as compared to other *Desulfovibrio* spp.: i) aerobic-type carbon monoxide dehydrogenase complex; ii) vacuolar-type ATP synthase complex; and iii) multisubunit Na+/H+ antiporter complex. Genes were assigned according to the predicted protein function. The unnamed coding regions are either hypothetical proteins or proteins of unknown function.

**Figure S2.** Interaction network of *D. gigas* proteins involved in cell size. Purple circles indicate central elements of the network. Yellow circles indicate elements with a fewer number of interactions. Blue lines show protein interactions common to several *Desulfovibrio* genus as retrieved by the STRING database .whereas red lines correspond to *D. gigas* specific interactions.

**General genome information**

**Table S1. Number of coding regions (CDS´s) associated with the general COG functions**

| Code | | Value | | Description | |
| --- | --- | --- | --- | --- | --- |
| Information Storage and Processing | | | | |  |
| K | 86 | | Transcription | |  |
| J | 148 | | Translation, ribosomal structure and biogenesis | |  |
| L | 104 | | Replication, recombination and repair | |  |
| B | 1 | | Chromatin structure and dynamics | |  |
| Metabolism | | | | |  |
| C | 184 | | Energy producing and conversion | |  |
| F | 55 | | Nucleotide transport and metabolism | |  |
| H | 97 | | Coenzyme transport and metabolism | |  |
| Q | 24 | | Secondary metabolites biosynthesis, transport and catabolism | |  |
| I | 36 | | Lipid transport and metabolism | |  |
| P | 112 | | Inorganic ion transport and metabolism | |  |
| G | 103 | | Carbohydrate transport and metabolism | |  |
| E | 208 | | Amino acid transport and metabolism | |  |
| Cellular processes and Signaling | | | | |  |
| U | 23 | | Intracellular trafficking, secretion and vesicular transport | |  |
| M | 143 | | Cell wall/membrane/envelope biogenesis | |  |
| V | 37 | | Defense mechanisms | |  |
| T | 263 | | Signal transduction mechanisms | |  |
| N | 131 | | Cell motility | |  |
| O | 91 | | Posttranslational modification, protein turnover, chaperones | |  |
| D | 32 | | Cell cycle control, cell division, chromosome partitioning | |  |
| Poorly characterized | | | | |  |
| S | 150 | | Function unknown | |  |
| R | 245 | | General function predicted only | |  |
| - | 999 | | No function | |  |

**Table S2 – Codon usage**

| **Codon** | **Aminoacid** | **Fraction** | **Number of AA** | **Codon** | **Aminoacid** | **Fraction** | **Number of AA** |
| --- | --- | --- | --- | --- | --- | --- | --- |
| GCA | Ala | 7.1% | 9240 | CCA | Pro | 5.3% | 3125 |
| **GCC** | **Ala** | **66.7%** | **86142** | **CCC** | **Pro** | **48.2%** | **28345** |
| GCG | Ala | 21.1% | 27259 | CCG | Pro | 38.5% | 22629 |
| GCT | Ala | 5.1% | 6604 | CCT | Pro | 8% | 4687 |
| **TGC** | **Cys** | **86.6%** | **13464** | CAA | Gln | 14.4% | 6158 |
| TGT | Cys | 13.4% | 2079 | **CAG** | **Gln** | **85.6%** | **36750** |
| **GAC** | **Asp** | **65.1%** | **36850** | AGA | Arg | 1.9% | 1389 |
| GAT | Asp | 34.9% | 19738 | AGG | Arg | 4.4% | 3241 |
| **GAA** | **Glu** | **50%** | **32497** | CGA | Arg | 3.8% | 2825 |
| **GAG** | **Glu** | **50%** | **32484** | **CGC** | **Arg** | **52.6%** | **38613** |
| **TTC** | **Phe** | **67.3%** | **26410** | CGG | Arg | 29.1% | 21408 |
| TTT | Phe | 32.7% | 12853 | CGT | Arg | 8.2% | 6018 |
| GGA | Gly | 6.9% | 5823 | AGC | Ser | 28.2% | 15018 |
| **GGC** | **Gly** | **67.4%** | **57198** | AGT | Ser | 4.3% | 2278 |
| GGG | Gly | 19.4% | 16443 | TCA | Ser | 2.9% | 1545 |
| GGT | Gly | 6.3% | 5453 | **TCC** | **Ser** | **44.6%** | **23742** |
| **CAC** | **His** | **60.9%** | **15731** | TCG | Ser | 15% | 7973 |
| CAT | His | 39.1% | 10114 | TCT | Ser | 5% | 2660 |
| ATA | Ile | 2.8% | 1320 | ACA | Thr | 6.1% | 3346 |
| **ATC** | **Ile** | **75.2%** | **35290** | **ACC** | **Thr** | **60.5%** | **33047** |
| ATT | Ile | 22% | 10342 | ACG | Thr | 29% | 15823 |
| AAA | Lys | 25.5% | 9599 | ACT | Thr | 4.4% | 2405 |
| **AAG** | **Lys** | **74.5%** | **28081** | GTA | Val | 2.6% | 2051 |
| CTA | Leu | 0.5% | 640 | GTC | Val | 24.2% | 19262 |
| CTC | Leu | 17.8% | 22100 | **GTG** | **Val** | **68.7%** | **54613** |
| **CTG** | **Leu** | **66.6%** | **82701** | GTT | Val | 4.5% | 3581 |
| CTT | Leu | 5.8% | 7257 | **TGG** | **Trp** | **100%** | **13642** |
| TTA | Leu | 0.4% | 547 | **TAC** | **Tyr** | **64.1%** | **15434** |
| TTG | Leu | 8.9% | 10925 | TAT | Tyr | 35.9% | 8629 |
| **ATG** | **Met** | **100%** | **28419** | TAA | * | 24.3% | 796 |
| **AAC** | **Asn** | **70.9%** | **18529** | TAG | * | 31% | 1015 |
| AAT | Asn | 29.1% | 7592 | **TGA** | ***** | **44.7%** | **1466** |

**MISCELLANEOUS**

**Table S3. Transposable elements**

| Encoded Protein | *D.gigas* ID | # AA | Gene symbol | CAI Index |
| --- | --- | --- | --- | --- |
| Putative transposase | 0060 | 114 |  | 0.372 |
| Putative transposase | 0061 | 118 |  | 0.348 |
| Putative transposase | 0492 | 176 |  | 0.422 |
| Transposase-like Mu | 0917 | 714 |  | 0.660 |
| Transposase, IS4 family protein | 1207 | 466 |  | 0.611 |
| Putative transposase | 1368 | 276 |  | 0.648 |
| Transposase IS3/IS911 family protein | 2017 | 41 |  | 0.698 |
| Integrase catalytic subunit | 2025 | 103 |  | 0.486 |
| Integrase catalytic region | 2394 | 732 |  | 0.686 |
| Transposase IS4 family protein | 2426 | 50 |  | 0.549 |
| Transposase IS4 family protein | 2446 | 353 |  | 0.617 |
| Putative transposase | 2457 | 105 |  | 0.565 |
| IS4 family transposase | 2643 | 436 |  | 0.577 |
| Putative transposase-like protein | 3314 | 159 |  | 0.346 |
| ISSoc4,transposase orfA | 3315 | 123 |  | 0.399 |
| Putative transposase | 3331 | 299 |  | 0.567 |
| Putative transposase-like protein | 3366 | 159 |  | 0.342 |

**Table S4. Selenocysteine-containing proteins**

| Encoded Protein | *D.gigas* ID | # AA | Gene symbol | CAI Index |
| --- | --- | --- | --- | --- |
| Selenocysteine-specific Translation Elongation Factor | 1858 | 643 | selB | 0.739 |
| L-seryl-tRNA Selenium Transferase | 1861 | 470 | selA | 0.744 |
| Conserved hypothetical protein | 2096 | 106 |  | 0.706 |
| HesB-like domain-containing protein | 2358 | 106 | hesB | 0.610 |
| Selenide, water Dikinase | 2804 | 325 | selD | 0.664 |
| DsrE family protein | 3368 | 106 |  | 0.706 |
| Cysteine Desulfurase / Selenocystein Lyase | 2344 | 383 | csdA | 0.669 |
| Selenium metabolism protein YedF | 2272 | 215 | yedF | 0.700 |
| Chain A, Tungsten Containing Formate Dehydrogenase | 1366 | 1012 | fdh IB | 0.752 |

**Table S5. CRISPR- associated proteins**

| Encoded Protein | *D.gigas* ID | # AA | Gene symbol | CAI Index |
| --- | --- | --- | --- | --- |
| CRISPR-associated Protein Cas1, YPEST subtype | 1866 | 326 | cas1 | 0.469 |
| CRISPR-associated Helicase Cas3 family | 1867 | 1109 | cas3 | 0.544 |
| CRISPR-associated Protein, Csy1 family | 1868 | 443 | csy1 | 0.549 |
| CRISPR-associated Protein, Csy2 family | 1869 | 309 | csy2 | 0.586 |
| CRISPR-associated Protein, Csy3 family | 1870 | 345 | csy3 | 0.525 |
| CRISPR-associated Protein, Csy4 family | 1871 | 186 | csy4 | 0.492 |
| Conserved hypothetical protein | 2447 | 337 |  | 0.440 |
| CRISPR-associated Helicase | 2448 | 982 | cas3 | 0.462 |
| CRISPR-associated Protein | 2449 | 475 | csb2 | 0.524 |
| CRISPR-associated Protein | 2450 | 404 | csb1 | 0.591 |

**Table S6. Chemotaxis proteins**

| Encoded Protein | *D.gigas* ID | # AA | Gene symbol | | CAI Index | |
| --- | --- | --- | --- | --- | --- | --- |
| CheB | 0024 | 374 | cheB | | 0.614 | |
| Hypothetical protein | 0023 | 648 |  | | 0.675 | |
| CheR | 0022 | 291 | cheR | | 0.662 | |
| ParA family protein | 0021 | 261 | parA | | 0.543 | |
| CheW | 0020 | 244 | cheW | | 0.686 | |
| CheY | 0019 | 372 | cheY | | 0.637 | |
| CheA | 0018 | 974 | cheA | | 0.707 | |
| Putative methyl-accepting chemotaxis protein | 3081 | 607 | mcp | | 0.696 | |
| Chemotaxis protein CheW | 3080 | 174 | cheW | | 0.680 | |
| Superoxide dismutase | 3082 | 130 | nlr | | 0.745 | |
| Chemotaxis protein CheW | 3083 | 168 | cheW | | 0.703 | |
| Methyl-accepting chemotaxis protein | 3084 | 649 | mcp | | 0.684 | |
| Anti-sigma-factor antagonist | 3079 | 102 |  | | 0.614 | |
| CheA signal transduction histidine kinase | 3078 | 700 |  | | 0.708 | |
| Methyl-accepting chemotaxis protein | 0120 | 599 | |  | | 0.698 |
| Methyl-accepting chemotaxis sensory transducer protein | 0381 | 672 | |  | | 0.715 |
| Methyl-accepting chemotaxis protein | 0398 | 676 | |  | | 0.693 |
| CheW protein | 0399 | 162 | |  | | 0.663 |
| Hypothetical protein | 0400 | 93 | |  | | 0.674 |
| Chemotaxis protein CheA | 0401 | 691 | |  | | 0.722 |
| Methyl-accepting chemotaxis protein | 0422 | 683 | |  | | 0.728 |
| CheW protein | 0423 | 162 | |  | | 0.698 |
| Methyl-accepting chemotaxis sensory transducer | 0498 | 710 | |  | | 0.744 |
| Methyl-accepting chemotaxis sensory transducer | 0662 | 572 | |  | | 0.695 |
| Response regulator receiver protein | 0820 | 130 | | cheY | | 0.602 |
| CheC, inhibitor of MCP methylation | 0821 | 218 | | cheC | | 0.721 |
| Chemotaxis protein CheA | 0822 | 101 | |  | | 0.661 |
| Methyl-accepting chemotaxis sensory transducer with Pas/Pac sensor | 0980 | 782 | |  | | 0.734 |
| Chemotaxis sensory transducer protein | 0986 | 813 | |  | | 0.723 |
| Chemotaxis protein | 1143 | 157 | |  | | 0.734 |
| Methyl-accepting chemotaxis sensory transducer | 1240 | 604 | |  | | 0.405 |
| Chemotaxis sensory transducer protein | 1382 | 779 | |  | | 0.693 |
| Methyl-accepting chemotaxis protein | 1490 | 600 | |  | | 0.757 |
| Methyl-accepting chemotaxis protein | 1602 | 544 | |  | | 0.645 |
| Chemotaxis protein CheW | 1665 | 158 | |  | | 0.712 |
| Methyl-accepting chemotaxis sensory transducer with Cache sensor | 1693 | 725 | |  | | 0.753 |
| CheW protein | 1942 | 166 | |  | | 0.677 |
| CheR-type MCP methyltransferase | 1943 | 477 | |  | | 0.663 |
| Chemotaxis protein CheW | 1944 | 212 | |  | | 0.594 |
| Methyl-accepting chemotaxis sensory transducer | 1945 | 583 | |  | | 0.736 |
| CheA signal transduction histidine kinase | 1946 | 776 | |  | | 0.657 |
| Response regulator receiver modulated CheB methylesterase | 1947 | 380 | |  | | 0.635 |
| Multi-sensor hybrid histidine kinase | 1948 | 1042 | |  | | 0.640 |
| Anti-sigma-factor antagonist | 1949 | 103 | |  | | 0.619 |
| CheD family protein | 1950 | 163 | |  | | 0.674 |
| Methyl-accepting chemotaxis sensory transducer | 2220 | 721 | |  | | 0.653 |
| Methyl-accepting chemotaxis sensory transducer with Cache sensor | 2263 | 572 | |  | | 0.666 |
| Multi-sensor hybrid histidine kinase | 2603 | 1195 | |  | | 0.659 |
| CheW protein | 2604 | 158 | |  | | 0.622 |
| Methyl-accepting chemotaxis sensory transducer | 2605 | 596 | |  | | 0.670 |
| Hypotetical protein | 2606 | 90 | |  | | 0.431 |
| CheD-like chemotaxis protein | 2639 | 160 | |  | | 0.546 |
| Methyl-accepting chemotaxis sensory transducer | 2675 | 679 | |  | | 0.642 |
| Methyl-accepting chemotaxis sensory transducer | 2707 | 583 | |  | | 0.682 |
| Methyl-accepting chemotaxis protein | 2714 | 576 | |  | | 0.567 |
| Methyl-accepting chemotaxis sensory transducer | 2743 | 676 | |  | | 0.686 |
| Chemotaxis protei | 2780 | 248 | |  | | 0.779 |
| Chemotaxis protein | 3056 | 167 | |  | | 0.646 |
| Response regulator receiver protein | 3057 | 156 | |  | | 0.599 |
| Methyl-accepting chemotaxis sensory transducer | 3240 | 459 | |  | | 0.604 |
| Chemotaxis protein histidine kinase CheA | 3241 | 701 | | cheA | | 0.609 |
| Alkaline phosphatase synthesis transcriptional regulatory protei | 3242 | 121 | |  | | 0.578 |
| Conserved hypothetical protein | 3243 | 100 | |  | | 0.578 |
| Conserved hypothetical protein | 3244 | 493 | |  | | 0.584 |
| Chemotaxis protein CheA | 3245 | 696 | |  | | 0.594 |
| Response regulator receiver protein | 3246 | 121 | |  | | 0.472 |
| Chemotaxis protein methyltransferase CheR | 3247 | 283 | |  | | 0.512 |
| Conserved hypothetical protein | 3248 | 220 | |  | | 0.497 |
| Chemotaxis-specific methylesterase CheB | 3249 | 359 | |  | | 0.590 |
| Chemoreceptor glutamine deamidase CheD | 3250 | 163 | |  | | 0.585 |
| CheW protein | 3251 | 546 | |  | | 0.637 |
| Methyl-accepting chemotaxis protein | 3252 | 650 | |  | | 0.631 |
| Response regulator receiver domain protein | 3253 | 129 | |  | | 0.506 |
| Methyl-accepting chemotaxis protein | 3422 | 879 | |  | | 0.725 |
| Response regulator with CheY-like receiver, AAA-type ATPase, and DNA-binding domains | 3423 | 123 | |  | | 0.664 |
| Chemotaxis sensory transducer | 3467 | 604 | |  | | 0.653 |
| Response regulator receiver protein | 3468 | 120 | |  | | 0.660 |
| CheA signal transduction histidine kinase | 3469 | 710 | |  | | 0.701 |
| Chemotaxis protein methyltransferase | 3470 | 281 | |  | | 0.650 |
| Response regulator receiver modulated CheB methylesterase | 3471 | 360 | |  | | 0.709 |

**GENERAL METABOLISM**

**Table S7. Response to oxygen**

| Encoded Protein | *D.gigas* ID | # AA | Gene symbol | CAI Index |
| --- | --- | --- | --- | --- |
| Putative superoxide dismutase | 1536 | 244 |  | 0.717 |
| Neelaredoxin, superoxide reductase / dismutase | 3082 | 130 | nlr | 0.745 |
| Catalase | 2858 | 502 | cat | 0.788 |
| Bacterioferritin | 2857 | 177 | bfr | 0.785 |
| Rubredoxin-like protein | 1167 | 71 | rub2 | 0.732 |
| Chain A, Chain B, Rubredoxin-oxygen Oxidoreductase | 1622 | 402 | roo | 0.802 |
| Chain A rubredoxin | 1624 | 52 | rd | 0.818 |
| Cytochrome bd Quinol Oxidase, subunit I | 1252 | 443 | cydA | 0.716 |
| Cytochrome bd Quinol Oxidase, subunit II | 1253 | 336 | cydB | 0.788 |
| Desulforedoxin | 3485 | 37 | dsr | 0.829 |
| Peroxiredoxin | 3518 | 222 | prxU | 0.738 |
| Rubrerythrin | 0750 | 165 | rbr | 0.761 |
| Rubrerythrin | 1055 | 156 | rbr | 0.684 |
| Rubrerythrin | 1714 | 162 |  | 0.710 |
| Coenzyme F390 synthetase | 2876 | 421 |  | 0.833 |
| Coenzyme F390 synthetase | 3140 | 441 |  | 0.784 |
| Coenzyme F390 synthetase-like | 0910 | 433 |  | 0.647 |

**Table S8. Nitrogen metabolism**

| Encoded Protein | *D.gigas* ID | # AA | Gene symbol | CAI Index |
| --- | --- | --- | --- | --- |
| Putative Nitrogenase Cofactor Biosynthesis Protein NifB | 1271 | 423 |  | 0.810 |
| Putative Nitrogenase | 1272 | 471 |  | 0.770 |
| Putative Nitrogenase MoFe Cofactor Biosynthesis Protein | 1273 | 475 |  | 0.796 |
| Putative Nitrogenase Cofactor Biosynthesis Protein NifB | 1275 | 403 |  | 0.764 |
| Putative Nitrogenase Molybdenum-iron Protein, beta chain | 1276 | 461 |  | 0.805 |
| Putative Nitrogenase Molybdenum-iron Protein, subunit alpha | 1277 | 555 |  | 0.824 |
| Putative Nitrogen Regulatory Protein P-II | 1278 | 126 |  | 0.746 |
| Putative Nitrogen Regulatory Protein P-II | 1279 | 118 |  | 0.693 |
| Nitrogenase Reductase | 1280 | 274 | nifH | 0.767 |
| Putative Nitrogen Regulatory Protein P-II | 2553 | 112 | glnB-1 | 0.790 |
| Putative Ammonium Transporter | 2554 | 402 | amt | 0.654 |
| Putative Nitrate Reductase | 0241 | 698 |  | 0.732 |
| Putative Nitrate Reductase | 1101 | 640 |  | 0.724 |
| Putative Nitrate Reductase | 1195 | 705 |  | 0.710 |
| Putative Transcriptional Regulator, NifA, Fis Family | 1208 | 515 | norR2L | 0.719 |
| Putative Fis family NifA subfamily Transcriptional Regulator | 0080 | 525 | norR1L | 0.743 |
| Putative Cytochrome c Nitrite Reductase, small subunit | 1513 | 143 | nrfH | 0.754 |
| Putative Nitrite Reductase | 1514 | 488 | nrfA | 0.780 |
| Putative Hydroxylamine Reductase | 1496 | 545 | hcp | 0.817 |
| Putative cAMP-binding protein | 1495 | 224 | hcpR | 0.748 |
| Putative Nitroreductase | 0813 | 174 |  | 0.720 |
| Putative Nitroreductase | 1466 | 305 |  | 0.739 |
| Putative Nitroreductase | 2864 | 169 |  | 0.652 |
| Putative Carbamoyl-phosphate Synthase, large subunit | 0189 | 1082 |  | 0.816 |
| Putative Carbamoyl-phosphate Synthase, small subunit | 0743 | 383 | carA | 0.782 |
| Putative Ornithine Carbamoyltransferase | 2162 | 301 | argF | 0.717 |
| Putative Argininosuccinate Synthase | 2161 | 403 | argG | 0.820 |
| Putative Argininosuccinate Lyase | 2160 | 462 |  | 0.732 |
| Putative Glutamate Synthase | 2742 | 507 |  | 0.740 |
| Putative Glutamate Synthase (NADPH), homotetrameric | 1689 | 481 | nfnB | 0.769 |
| Putative Glutamine Synthetase, type I | 1150 | 448 |  | 0.764 |

**Table S9. Transcriptional factors Sigma 54**

| Encoded Protein | *D.gigas* ID | # AA | Gene symbol | CAI Index |
| --- | --- | --- | --- | --- |
| Putative Sigma-54 dependent Transcriptional Regulator/Response Regulator | 0875 | 458 |  | 0.766 |
| Putative two component, Sigma54 specific, Transcriptional Regulator, Fis family | 0999 | 478 |  | 0.734 |
| Putative Fis family two component, Sigma-54 specific, Transcriptional Regulator | 1046 | 453 |  | 0.796 |
| Putative two component, Sigma54 specific, Fis family Transcriptional Regulator | 1069 | 486 |  | 0.753 |
| Putative Sigma-54 Factor Interaction domain-containing Protein | 1255 | 468 |  | 0.714 |
| Putative two component, Sigma54 specific, Transcriptional Regulator | 1424 | 507 |  | 0.664 |
| Putative Fis family Sigma-54 specific activator | 1482 | 338 |  | 0.663 |
| Putative two component Sigma-54 specific Transcriptional Regulator | 1580 | 470 |  | 0.777 |
| Putative Fis family two component Sigma-54 specific Transcriptional Regulator | 1653 | 473 |  | 0.669 |
| Putative Fis family two component Sigma-54 specific Transcriptional Regulator | 1941 | 479 |  | 0.726 |
| Putative ECF subfamily RNA Polymerase Sigma-24 | 2673 | 202 |  | 0.633 |
| Putative Sigma 54 interacting domain Protein | 3035 | 844 |  | 0.512 |
| Putative PAS modulated Sigma54 specific Transcriptional Regulator, Fis family | 1702 | 441 |  | 0.632 |

**Table S10. Sulfate metabolism**

| Encoded Protein | *D.gigas* ID | # AA | Gene symbol | CAI Index |
| --- | --- | --- | --- | --- |
| Adenylylsulfate Reductase B subunit | 0458 | 167 | aprB | 0.757 |
| Adenylylsulfate Reductase A subunit | 0457 | 666 | aprA | 0.734 |
| Adenylylsulfate Kinase | 1831 | 196 | cycC | 0.646 |
| Desulforedoxin | 3485 | 37 | dsr | 0.829 |
| Cobyrinic Acid A,C-diamide Synthase | 0687 | 487 |  | 0.679 |
| Dissimilatory Sulfite Reductase B | 0688 | 75 | dsrD | 0.634 |
| Dissimilatory Sulfite Reductase I (Dsri) B subunit | 0689 | 386 | dsrB | 0.738 |
| Dissimilatory Sulfite Reductase I (Dsri) A subunit | 0691 | 437 | dsrA | 0.723 |
| Dissimilatory Sulfite Reductase, gamma subunit | 1681 | 105 | dsrC | 0.735 |
| DsrE family protein | 1238 | 118 |  | 0.759 |
| Nitrite and Sulfite Reductase, 4Fe-4S region | 3367 | 217 |  | 0.772 |
| DsrE family protein | 3368 | 106 |  | 0.706 |
| Phosphoadenosine Phosphosulfate Reductase | 3007 | 273 | paps | 0.576 |
| Sulfate Adenylyltransferase | 0460 | 426 | sat | 0.778 |
| DnaJ-like Protein | 0854 | 132 |  | 0.752 |
| Protein of unknown function | 0855 | 211 |  | 0.670 |
| Mammalian cell entry domain-containing protein | 0856 | 310 |  | 0.671 |
| Sulfate-transporting ATPase | 0857 | 259 |  | 0.711 |
| Protein of unknown function | 0858 | 250 |  | 0.678 |
| Putative Sulfate Transport Protein CysZ | 0923 | 214 |  | 0.740 |
| Putative Sulfate Transport Protein CysZ | 2397 | 205 |  | 0.599 |
| Sulfate Permease family Protein | 0373 | 561 | sulP | 0.702 |

**Central AND ENERGY MetabolisM**

**Table S11. Pentose Phosphate Pathway**

| Encoded Protein | *D.gigas* ID | # AA | Gene symbol | CAI Index |
| --- | --- | --- | --- | --- |
| Transketolase | 1348 | 666 | tkt | 0.761 |
| Glucose-6-Phosphate Isomerase | 2341 | 552 | gpi | 0.708 |
| Ribulose-Phosphate 3-Epimerase | 1351 | 226 | rpe | 0.740 |
| Translaldolase | 2275 | 220 | tal | 0.666 |
| 6-Phosphogluconate Dehydrogenase, Decarboxylating | 1384 | 301 | gnd | 0.717 |
| Glucose-6-Phosphate 1-Dehydrogenase | 1385 | 523 | zwf | 0.679 |
| 6-Phosphogluconolactonase | 1386 | 242 | pgl | 0.578 |
| Ribose-5-Phosphate Isomerase A | 1387 | 236 | rpi | 0.662 |

**Table S12. Beta Oxidation**

| Encoded Protein | *D.gigas* ID | # AA | Gene symbol | CAI Index |
| --- | --- | --- | --- | --- |
| N-acetyltransferase GCN5 | 2273 | 161 |  | 0.518 |
| Acyl-CoA Dehydrogenase domain-containing protein | 1478 | 689 |  | 0.756 |
| Electron-transferring-flavoprotein Dehydrogenase | 1479 | 618 |  | 0.762 |
| Electron Transfer Flavoprotein, Beta subunit | 1480 | 268 | etfB | 0.711 |
| Electron Transfer Flavoprotein, Alpha subunit | 1481 | 339 | etfA | 0.717 |

**Table S13. Embden-Meyerhof-Parnas Pathway**

| Encoded Protein | *D.gigas* ID | # AA | Gene symbol | CAI Index |
| --- | --- | --- | --- | --- |
| Enolase (phosphopyruvate hydratase) | 0704 | 441 | eno | 0.822 |
| Fructose-bisphosphate Aldolase | 0591 | 267 | fbaB | 0.702 |
| Putative Phospho-2-dehydro-3-deoxyheptonate Aldolase | 0592 | 267 | fbaB2 | 0.722 |
| Fructose-bisphosphate Aldolase | 1026 | 257 | fbaB1 | 0.724 |
| Fructose-1,6-bisphosphatase | 2524 | 349 | fbp | 0.696 |
| Type I Glyceraldehyde-3-phosphate Dehydrogenase | 2188 | 333 | gap | 0.766 |
| Glyceraldehyde-3-phosphate Dehydrogenase, type I | 2546 | 338 | gap1 | 0.755 |
| Glucokinase | 1383 | 327 | gck | 0.654 |
| Glycogen Phosphorylase | 2153 | 855 | glgP | 0.731 |
| Phosphoglycerate Mutase 1 family | 1753 | 248 | gpmA | 0.774 |
| 2,3-Bisphosphoglycerate-independent Phosphoglycerate Mutase | 3489 | 532 | gpmB | 0.714 |
| PEP Synthase | 2938 | 848 |  | 0.791 |
| Diphosphate--fructose-6-phosphate 1-Phosphotransferase | 2195 | 456 | pfkA | 0.747 |
| Phosphoglycerate Kinase | 1347 | 391 | pgk | 0.784 |
| Phosphoglucomutase, Alpha-D-glucose Phosphate-specific | 0235 | 549 | pgm | 0.785 |
| PTS System Mannose/fructose/sorbose IID component family protein | 3110 | 462 |  | 0.711 |
| Phosphocarrier protein HPr | 3111 | 162 | ptsH | 0.758 |
| Phosphoenolpyruvate-protein Phosphotransferase | 3112 | 594 | ptsA | 0.749 |
| PTS System Sorbose subfamily transporter subunit IIB | 2413 | 156 |  | 0.741 |
| PTS System Fructose IIA component family protein | 2414 | 149 |  | 0.655 |
| PTS IIA-like Nitrogen-regulatory Protein PtsN | 2416 | 149 | ptsN | 0.640 |
| Pyruvate Kinase | 0179 | 483 | pyk | 0.726 |
| Triose-phosphate Isomerase | 0332 | 249 | tpi | 0.569 |

**Table S14. Entner-Doudoroff Pathway**

| Encoded Protein | *D.gigas* ID | # AA | Gene symbol | CAI Index |
| --- | --- | --- | --- | --- |
| Dihydroxy-Acid Dehydratase | 0845 | 555 | edd | 0.806 |
| Glucose-6-phosphate 1-Dehydrogenase | 1385 | 523 | zwf | 0.679 |
| Glucokinase | 1383 | 327 | gck | 0.654 |

**Table S15. TCA Cycle**

| Encoded Protein | *D.gigas* ID | # AA | Gene symbol | CAI Index |
| --- | --- | --- | --- | --- |
| Aconitate Hydratase | 0463 | 651 | aco | 0.788 |
| Fumarate Hydratase | 1571 | 278 | fumA | 0.821 |
| Fe-S Type, Tartrate/fumarate subfamily Hydro-lyase subunit alpha | 1573 | 181 | fumC | 0.790 |
| Citrate Synthase I | 1379 | 436 | gltA | 0.737 |
| Isocitrate Dehydrogenase | 1851 | 382 | icd | 0.806 |
| Malic Protein NAD-binding protein | 1574 | 437 | mdh | 0.803 |

**Table S16. Fumarate Metabolism**

| Encoded Protein | *D.gigas* ID | # AA | Gene symbol | CAI Index |
| --- | --- | --- | --- | --- |
| Fumarate Reductase Respiratory Complex | 1568 | 218 | fdrC | 0.643 |
| Fumarate Reductase, Flavoprotein subunit | 1569 | 627 | fdrA | 0.810 |
| Fumarate Reductase, Iron-sulfur subunit | 1570 | 264 | fdrB | 0.744 |
| Fumarate reductase Respiratory Complex, transmembrane subunit | 0826 | 224 | fdrCII | 0.686 |
| Fumarate Reductase, Flavoprotein subunit | 0827 | 615 | fdrAII | 0.811 |
| Fumarate Reductase, Iron-sulfur subunit | 0828 | 255 | fdrBII | 0.792 |

**Table S17. WoodLjungdahl Pathway**

| Encoded Protein | *D.gigas* ID | # AA | Gene symbol | CAI Index |
| --- | --- | --- | --- | --- |
| Acyl-CoA Synthetase (NDP forming) | 0571 | 905 | acsA | 0.751 |
| Homocysteine S-Methyltransferase | 3461 | 816 | acsE | 0.709 |
| Aerobic-type Carbon Monoxide Dehydrogenase, small subunit CoxS/CutS-like protein | 3143 | 228 | coxS | 0.701 |
| Aerobic-type Carbon Monoxide Dehydrogenase, large subunit CoxL/CutL-like protein | 3144 | 782 | coxL | 0.737 |
| Aerobic-type Carbon Monoxide Dehydrogenase, middle subunit CoxM/CutM-like protein | 3145 | 325 | coxM | 0.674 |
| Bifunctional 5,10-Methylene-tetrahydrofolate Dehydrogenase/ 5,10-Methylene-tetrahydrofolate Cyclohydrolase | 0702 | 295 | folD | 0.726 |
| 5,10-Methylenetetrahydrofolate Reductase | 0415 | 304 | metF | 0.736 |
| Cobalamin B12-Binding domain Protein | 2600 | 229 | mtsB | 0.599 |

**Table S18. Alcohol metabolism**

| Encoded Protein | *D.gigas* ID | # AA | Gene symbol | CAI Index |
| --- | --- | --- | --- | --- |
| Iron-containing Alcohol Dehydrogenase | 0712 | 400 |  | 0.695 |
| Iron-containing Alcohol Dehydrogenase | 1044 | 380 |  | 0.729 |
| Iron-containing Alcohol Dehydrogenase | 1047 | 393 |  | 0.749 |
| Zinc-containing Alcohol Dehydrogenase | 2585 | 323 |  | 0.657 |
| Zinc-containing Alcohol Dehydrogenase | 3525 | 425 |  | 0.688 |
| Aldehyde Dehydrogenase | 0746 | 477 | ald | 0.772 |

**Table S19. Lactate Metabolism**

| Encoded Protein | *D.gigas* ID | # AA | Gene symbol | CAI Index | |
| --- | --- | --- | --- | --- | --- |
| D-Lactate Dehydrogenase | 1422 | 460 | ldh | 0.833 |  |
| Glycolate Oxidase subunit GlcD | 3110 | 462 |  | 0.711 |  |
| FMN-dependent Alpha-hydroxy Acid Dehydrogenase | 1415 | 345 |  | 0.724 |  |
| Conserved hypothetical protein | 1416 | 721 |  | 0.750 |  |
| Lactate Utilization Protein B/C | 1417 | 208 |  | 0.741 |  |
| Putative L-Lactate Transport | 1423 | 566 |  | 0.749 |  |

**Table S20. Formate Metabolism**

| Encoded Protein | *D.gigas* ID | # AA | Gene symbol | CAI Index |
| --- | --- | --- | --- | --- |
| Chain B, Tungsten Containing Formate Dehydrogenase | 1364 | 245 | fdhB | 0.745 |
| Chain A, Tungsten Containing Formate Dehydrogenase | 1366 | 1012 | fdh A | 0.752 |
| Formate Dehydrogenase, alpha subunit | 3334 | 1009 | fdnG | 0.771 |
| Putative Fe-S-cluster-containing Hydrogenase component protein | 3335 | 247 |  | 0.701 |
| Formate Dehydrogenase accessory protein | 0759 | 315 | fdhE | 0.739 |
| Formate Dehydrogenase subunit FdhD | 0760 | 236 | fdhD | 0.667 |
| Putative Formate Dehydrogenase, formation protein FdhE1 | 3336 | 303 | fdhEI | 0.636 |

**Table S21. Oxidation of pyruvate to acetyl-CoA and acetate formation**

| Encoded Protein | *D.gigas* ID | # AA | Gene symbol | CAI Index |
| --- | --- | --- | --- | --- |
| Aldehyde Ferredoxin Oxidoreductase | 1447 | 561 |  | 0.633 |
| Aldehyde Ferredoxin Oxidoreductase | 3127 | 579 |  | 0.784 |
| Ferredoxin I | 1020 | 62 |  | 0.741 |
| Ferredoxin II | 0418 | 59 |  | 0.700 |
| Conserved hypothetical protein | 2139 | 86 |  | 0.749 |
| Ferrodoxin-like protein | 0869 | 81 | vorC | 0.720 |
| 2-Ketoisovalerate Ferredoxin Reductase | 0870 | 353 | vorA | 0.768 |
| Thiamine pyrophosphate binding domain-containing protein | 0871 | 270 | vorB | 0.752 |
| 2-Oxoacid:ferredoxin Oxidoreductase, gamma subunit | 0872 | 187 | vorG | 0.784 |
| Indolepyruvate Ferredoxin Oxidoreductase | 0081 | 201 |  | 0.657 |
| Indolepyruvate Ferredoxin Oxidoreductase subunit alpha | 0082 | 632 | iorA | 0.707 |
| Pyruvate-Ferredoxin Oxidoreductase | 0996 | 1213 | poR | 0.805 |
| Pyruvate Ferredoxin/Flavodoxin Oxidoreductase subunit beta | 1712 | 283 | porB | 0.679 |
| Pyruvate Flavodoxin/Ferredoxin Oxidoreductase domain protein | 1713 | 563 | porA | 0.642 |
| Biotin/Acetyl-CoA-Carboxylase Ligase | 3401 | 326 | pycA | 0.556 |
| Pyruvate Carboxylase | 3402 | 1268 | pycB | 0.754 |
| Pyruvate, water Dikinase., Phosphoenolpyruvate--protein Phosphotransferase | 2250 | 1208 | ppdk | 0.789 |
| Pyruvate, water Dikinase | 3042 | 883 |  | 0.721 |
| Phosphoenolpyruvate Synthase/Pyruvate Phosphate Dikinase | 3046 | 882 |  | 0.764 |
| Pyruvate, water Dikinase | 2942 | 841 |  | 0.760 |

**Table S22. ATP synthesis**

| Encoded Protein | *D.gigas* ID | # AA | Gene symbol | CAI Index |
| --- | --- | --- | --- | --- |
| ATP synthase F0F1 subunit epsilon | 0648 | 133 | atpC | 0.616 |
| ATP synthase F1, beta subunit | 0649 | 471 | atpD | 0.728 |
| F0F1 ATP synthase subunit gamma | 0650 | 293 | atpG | 0.602 |
| F0F1 ATP synthase subunit alpha | 0651 | 502 | atpA | 0.752 |
| ATP synthase F0F1 subunit delta | 0652 | 183 | atpH | 0.591 |
| H+transporting two-sector ATPase subunit B/B' | 0653 | 189 | atpF | 0.604 |
| H+transporting two-sector ATPase subunit B/B' | 0654 | 138 | atpF1 | 0.548 |
| ATP synthase protein I | 1499 | 248 | atpI | 0.499 |
| ATP synthase I | 1500 | 156 | uncI | 0.521 |
| ATP synthase F0 subunit alpha | 1501 | 234 | atpB | 0.671 |
| ATP synthase F0, C subunit | 1502 | 110 | atpE | 0.677 |
| V-type ATP synthase subunit K | 3061 | 162 |  | 0.698 |
| V-type ATPase 116 kDa subunit | 3062 | 622 |  | 0.672 |
| H(+)-transporting ATP synthase, vacuolar type, subunit D | 3063 | 205 |  | 0.721 |
| V-type ATP synthase subunit B | 3064 | 430 |  | 0.776 |
| V-type ATP synthase subunit A | 3065 | 583 |  | 0.768 |
| Two-sector ATPase, V(1) subunit E | 3067 | 214 |  | 0.639 |

**Table S23. Cytochromes**

| Encoded Protein | *D.gigas ID* | # AA | Gene symbol | CAI Index |
| --- | --- | --- | --- | --- |
| Cytochrome bd Quinol Oxidase subunit I | 1252 | 443 | cydA | 0.716 |
| Cytochrome bd Quinol Oxidase subunit II | 1253 | 336 | cydB | 0.788 |
| Cytochrome c class III | 0326 | 133 |  | 0.492 |
| Cytochrome c3 | 0144 | 137 | cyc | 0.767 |
| Di-Tetraheme Cytochrome C3 | 1464 | 112 |  | 0.834 |
| Cytochrome c554 | 0380 | 157 |  | 0.689 |
| Respiratory Nitrite Reductase specific menaquinol--cytochrome-c reductase (NrfH) precursor | 1513 | 143 | nrfH | 0.754 |
| Nitrite Reductase (cytochrome, ammonia-forming) | 1514 | 488 | nrfA | 0.780 |
| Conserved protein of unknown function | 2209 | 47 |  | 0.619 |
| Cytochrome c assembly protein | 2210 | 225 | ccmC | 0.636 |
| ccmB family protein | 2211 | 224 | ccmB | 0.554 |
| Heme exporter protein CcmA | 2212 | 226 | ccmA | 0.692 |
| Cytochrome C assembly protein | 2213 | 635 | ccmF | 0.714 |
| Conserved hypothetical protein | 2214 | 458 |  | 0.643 |
| Cytochrome c-type biogenesis protein CcmE | 2216 | 140 | ccmE | 0.682 |
| Protein of unknown function | 2217 | 360 |  | 0.700 |

**Table S24. Hydrogenases**

| Encoded Protein | D.gigas ID | # AA | Gene symbol | CAI Index |
| --- | --- | --- | --- | --- |
| hynD | 2259 | 82 | hynD | 0.695 |
| hynC | 2260 | 165 | hynC | 0.756 |
| hynB | 2261 | 551 | hynB | 0.802 |
| hynA | 2262 | 288 | hynA | 0.717 |
| echA | 0034 | 647 | echA | 0.642 |
| echB | 0035 | 284 | echB | 0.716 |
| echC | 0036 | 147 | echC | 0.727 |
| echD | 0037 | 125 | echD | 0.704 |
| echE | 0038 | 358 | echE | 0.775 |
| echF | 0039 | 123 | echF | 0.795 |
| (NiFe) Hydrogenase maturation protein HypF | 0896 | 818 | hypF | 0.679 |
| Hydrogenase expression/formation protein HypD | 1098 | 372 | hypD | 0.764 |
| Hydrogenase expression/formation protein HypE | 1099 | 340 | hypE | 0.706 |
| Hydrogenase accessory protein HypB | 2238 | 219 | hypB | 0.729 |
| HypA | 2239 | 121 | hypA | 0.760 |

**ENERGY CONSERVATION**

**Table S25. Membranar Energy Complexes**

| Encoded Protein | *D.gigas* ID | # AA | Gene symbol | CAI Index |
| --- | --- | --- | --- | --- |
| Hdr-like Menaquinol Oxidoreductase Cytochrome b-like subunit | 2334 | 338 | dsrM | 0.658 |
| Cytoplasmic, binds 2 (4Fe-4S) | 2335 | 542 | dsrK | 0.758 |
| Periplasmic (Sec) Triheme Cytochrome c | 2336 | 127 | dsrJ | 0.731 |
| Periplasmic (Tat), binds 2(4Fe-4S) | 2337 | 268 | dsrO | 0.798 |
| Polysulphide Reductase NrfD | 2338 | 386 | dsrP | 0.756 |
| Sixteen Heme Cytochrome | 0715 | 559 | hmcA | 0.697 |
| 4Fe-4S Ferredoxin | 0716 | 364 | hmcB | 0.735 |
| HMC redox complex, integral membrane protein HmcC | 0717 | 416 | hmcC | 0.796 |
| protein HmcD | 0718 | 48 | hmcD | 0.622 |
| HMC redox complex, integral membrane protein HmcE | 0719 | 225 | hmcE | 0.744 |
| protein HmcF | 0720 | 470 | hmcF | 0.796 |
| NAD(P)H-quinone Oxidoreductase subunit 3 | 0242 | 125 | nuoA | 0.568 |
| NADH-quinone Oxidoreductase subunit B | 0243 | 192 | nuoB | 0.670 |
| NADH:ubiquinone Oxidoreductase 27 kD subunit | 0244 | 568 | nuoC/D | 0.764 |
| NADH Dehydrogenase (quinone) | 0245 | 328 | nuoH | 0.670 |
| NADH:ubiquinone Oxidoreductase chain I-like protein | 0246 | 218 | nuoI | 0.681 |
| NADH-ubiquinone/plastoquinone Oxidoreductase chain 6 | 0247 | 172 | nuoJ | 0.624 |
| NADH-quinone Oxidoreductase subunit K 1 | 0248 | 113 | nuoK | 0.570 |
| NADH/Ubiquinone/plastoquinone (Complex I) | 0249 | 492 | nuoL | 0.747 |
| hypothetical protein B193_0141 | 0250 | 90 |  | 0.707 |
| Monovalent cation/H+ Antiporter subunit D | 0251 | 613 | nuoN | 0.760 |
| Proton-translocating NADH-quinone Oxidoreductase subunit M | 0253 | 521 | nuoM | 0.737 |
| NADH Dehydrogenase (quinone) | 0254 | 475 |  | 0.745 |
| 4Fe-4S Ferredoxin | 0255 | 169 |  | 0.816 |
| Permease | 0256 | 378 | nuoP | 0.750 |
| Conserved hypothetical protein | 0257 | 219 |  | 0.739 |
| Multicomponent Na+/H+ Antiporter subunit E | 2843 | 164 | mnhE | 0.705 |
| Multiple Resistance and pH Regulation protein F | 2844 | 100 | mnhF | 0.660 |
| Multicomponent Na+/H+ Antiporter subunit G | 2845 | 122 | mnhG | 0.572 |
| Conserved hypothetical protein | 2846 | 81 |  | 0.602 |
| Putative Monovalent cation/H+ Antiporter subunit B | 0247 | 266 | mnhB | 0.588 |
| Na(+)/H(+) Anitporter subunit MhnC | 0248 | 129 | mnhC | 0.583 |
| NADH/ubiquinone/plastoquinone | 2849 | 465 |  | 0.662 |
| NADH Dehydrogenase (quinone) | 2850 | 513 | mnhA | 0.561 |
| Conserved hypothetical protein | 2851 | 81 |  | 0.492 |
| Putative Monovalent cation/H+ Antiporter subunit D | 2852 | 598 | mnhD | 0.654 |
| Crp family Transcriptional Regulator | 2853 | 164 |  | 0.615 |
| Hydrogenase, b-type Cytochrome subunit | 0374 | 202 | ohcC | 0.724 |
| Cytochrome c family protein | 0375 | 545 | ohcA | 0.735 |
| Iron-sulfur Cluster-binding Protein | 0376 | 157 |  | 0.606 |
| 4Fe-4S Ferredoxin | 0377 | 323 | ohcB | 0.666 |
| QmoD protein | 0453 | 246 | qmoD | 0.674 |
| Heterodisulfide Reductase | 0454 | 393 | qmoC | 0.734 |
| Quinone-interacting Membrane-bound Oxidoreductase | 0455 | 768 | qmoB | 0.768 |
| Heterodisulfide Reductase | 0456 | 411 | qmoA | 0.691 |
| Quinone-interacting Membrane-bound Oxidoreductase complex subunit C | 2765 | 402 |  | 0.694 |
| Conserved hypothetical protein | 2766 | 228 |  | 0.762 |
| Polysulfide Reductase NrfD | 0130 | 411 | qrcD | 0.774 |
| Molybdopterin Oxidoreductase, Iron-sulfur Cluster-binding subunit | 0131 | 266 | qrcC | 0.691 |
| Molybdopterin Oxidoreductase | 0132 | 693 | qrcB | 0.706 |
| Cytochrome C | 0133 | 212 | qrcA | 0.623 |
| CheY-like receiver, AAA-type ATPase, and DNA-binding domain containing response regulator | 1424 | 507 |  | 0.664 |
| ApbE family Lipoprotein | 1425 | 344 | rnfF | 0.750 |
| 4Fe-4S Ferredoxin | 1426 | 293 | rnfB | 0.772 |
| RnfABCDGE type electron transport complex subunit A | 1427 | 191 | rnfA | 0.658 |
| RnfABCDGE type electron transport complex subunit E | 1428 | 239 | rnfE | 0.657 |
| RnfABCDGE type electron transport complex subunit G | 1429 | 193 | rnfG | 0.773 |
| RnfABCDGE type electron transport complex subunit D | 1430 | 318 | rnfD | 0.671 |
| 4Fe-4S Ferredoxin | 1431 | 398 | rnfC | 0.730 |
| Cytochrome c family protein | 1432 | 245 |  | 0.710 |
| Transmembrane complex, Tetraheme Cytochrome c3 | 1698 | 137 | tmcA | 0.760 |
| Iron-sulfur binding Protein | 1699 | 444 | tmcB | 0.770 |
| Conserved hypothetical protein | 1700 | 219 | tmcC | 0.647 |
| TmC complex protein, subunit D | 1701 | 419 | tmcD | 0.695 |

**Table S26. Nfn complex**

| Encoded Protein | *D.gigas* ID | # AA | Gene symbol | CAI Index |
| --- | --- | --- | --- | --- |
| FAD-dependent Pyridine Nucleotide-disulfide Oxidoreductase | 1688 | 458 |  | 0.650 |
| Oxidoreductase | 1689 | 481 | nfnB | 0.769 |
| Ferredoxin-NADP(+) Reductase subunit alpha | 1690 | 282 | nfnA | 0.736 |
| Oxidoreductase | 1577 | 475 |  | 0.768 |
| Ferredoxin-NADP Reductase | 1578 | 261 |  | 0.717 |

**Table S27. Hdr-like proteins**

| Encoded Protein | *D.gigas* ID | # AA | Gene symbol | CAI Index |
| --- | --- | --- | --- | --- |
| Heterodisulfide Reductase subunit C | 1048 | 197 | hdrC | 0.711 |
| CoB--CoM Heterodisulfide Reductase | 1049 | 299 | hdrB | 0.769 |
| Heterodisulfide Reductase, subunit A | 1050 | 669 | hdrA | 0.786 |
| Methyl-viologen-reducing Hydrogenase delta subunit | 1051 | 149 | floxD | 0.765 |
| Coenzyme F420 Hydrogenase/Dehydrogenase, beta subunit | 1052 | 321 | floxC | 0.707 |
| Hydrogenase, putative | 1053 | 403 | floxB | 0.742 |
| Dihydroorotate Dehydrogenase, electron transfer subunit protein | 1054 | 277 | floxA | 0.778 |
| FAD linked Oxidase domain-containing Protein | 1343 | 1199 | hdrD | 0.749 |
| Iron-sulfur cluster-binding protein | 1416 | 721 |  | 0.750 |
| Iron-sulfur cluster-binding protein | 1421 | 426 |  | 0.798 |
| Fe-S Oxidoreductase | 3109 | 379 |  | 0.677 |
| Aldehyde Dehydrogenase, iron-sulfur subunit | 0904 | 762 |  | 0.673 |
