# Supplementary figures and images for: Genome sequence of the model sulfate reducer Desulfovibrio gigas: a comparative analysis within the Desulfovibrio genus
Source: Microbiologyopen. 2014 Jul 23;3(4):513–30. doi: 10.1002/mbo3.184 (PMC4287179; doi:10.1002/mbo3.184)

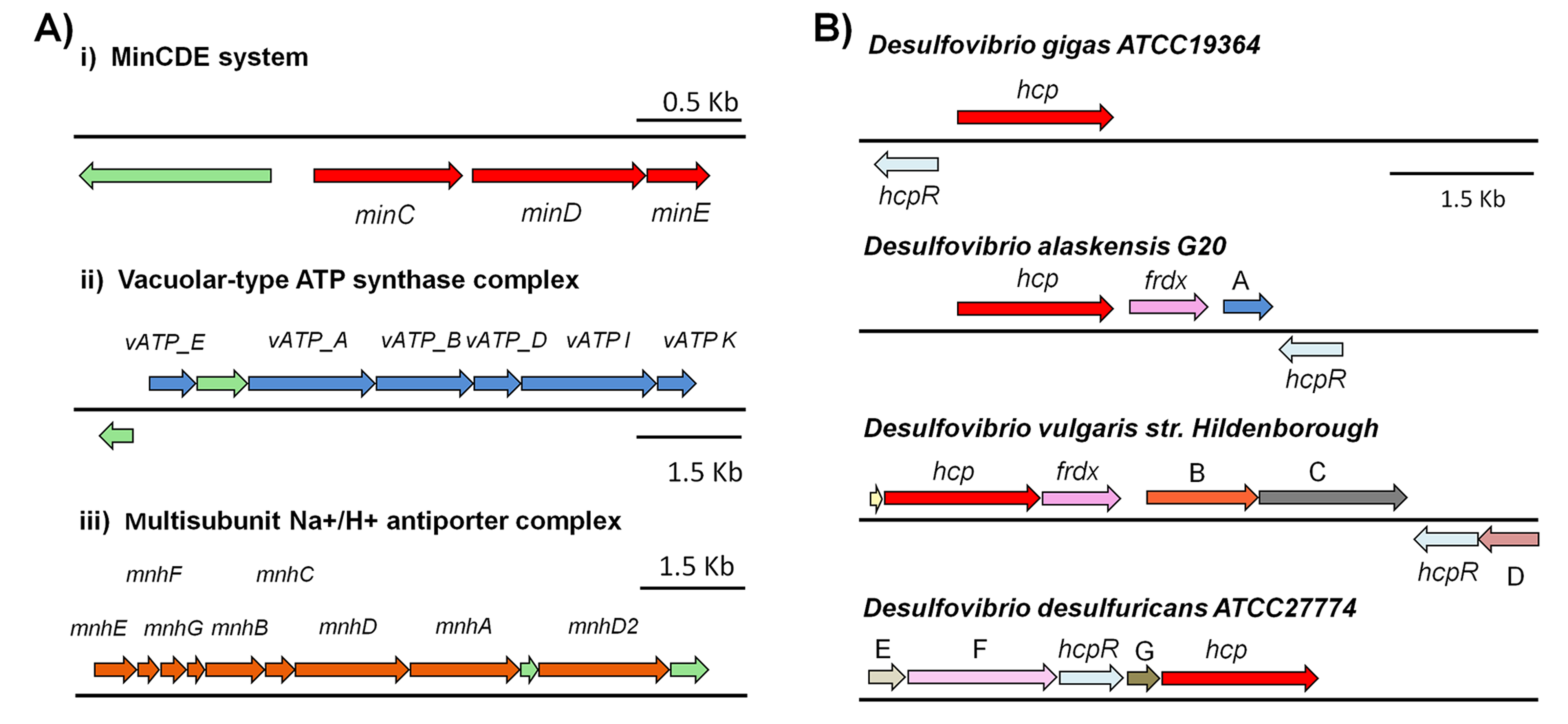

Supplement: Figure S2 — Interaction network of Desulfovibrio gigas proteins involved in cell size. Purple circles indicate central elements of the network. Yellow circles indicate elements with a fewer number of interactions. Blue lines show protein interactions common to several D. genus as retrieved by the STRING database,whereas red lines correspond to D. gigas specific interactions. [file mbo30003-0513-sd2.tif]

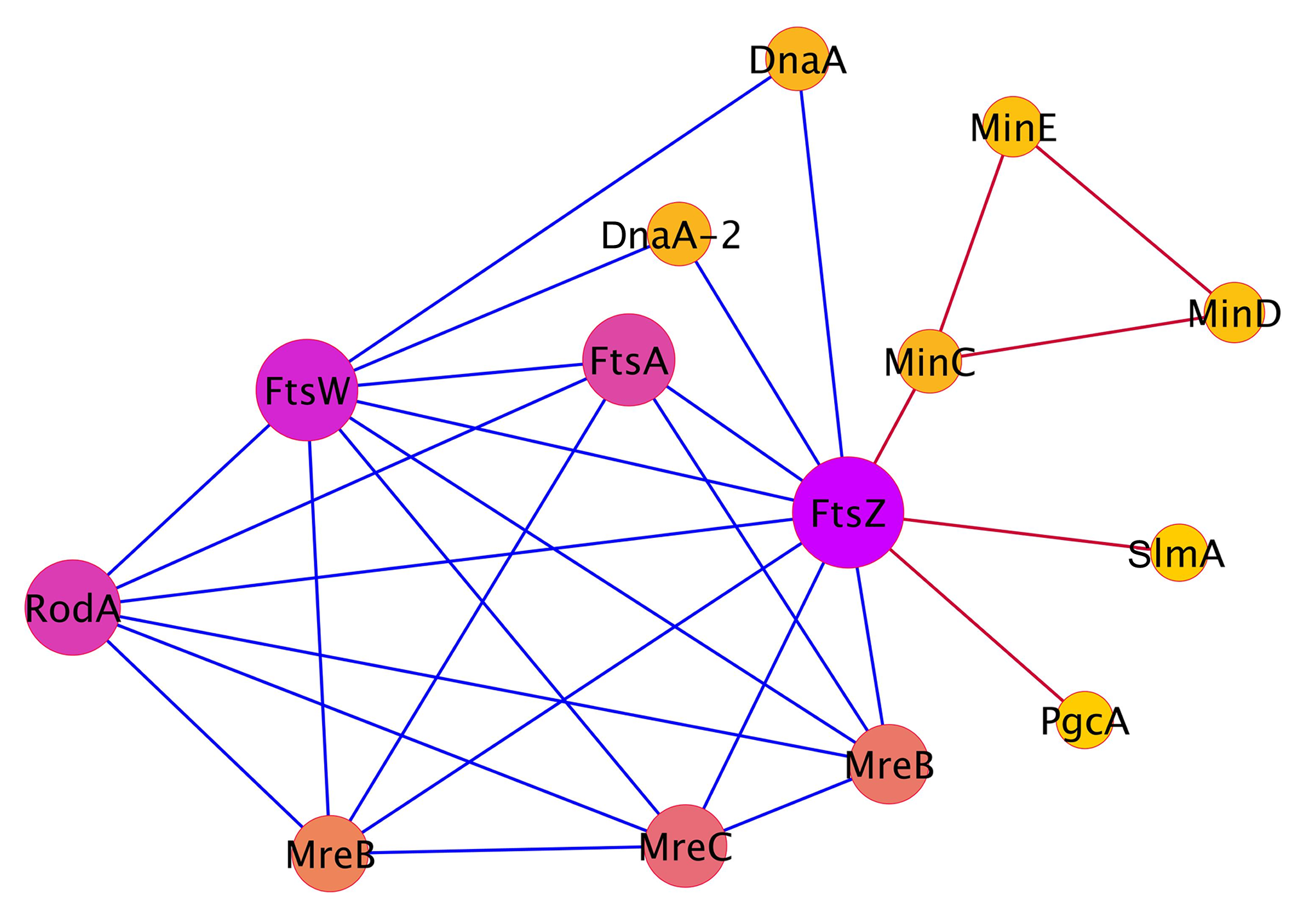

Supplement: Table S1 — COG functional groups. Table S2. Codon usage. Table S3. Transposable elements. Table S4. Selenocystein-containing proteins. Table S5. CRISPR proteins. Table S6. Chemotaxis proteins. Table S7. Response to oxygen. Table S8. Nitrogen metabolism. Table S9. Transcriptional factors sigma 54. Table S10. Sulfate metabolism. Table S11. Pentose phosphate pathway. Table S12. Beta oxidation. Table S13. Embden-Meyerhof-Parnas pathway. Table S14: Entner-Doudoroff pathway. Table S15. TCA cycle. Table S16. Fumarate metabolism. Table S17. WoodLjungahl pathway. Table S18. Alcohol metabolism. Table S19. Lactate metabolism. Table S20. Formate metabolism. Table S21. Oxidation of pyruvate to acetyl-CoA and acetate formation. Table S22. ATP synthesis. Table S23. Cytochromes. Table S24. Hydrogenases. Table S25. Membranar energy complexes. Table S26. Nfn complexes. Table S27. Hdr-like proteins. [file mbo30003-0513-sd3.tif]
